# Machine Learning Models for Predicting Molecular Diffusion in Metal–Organic Frameworks Accounting for the Impact of Framework Flexibility

**DOI:** 10.1021/acs.chemmater.3c02321

**Published:** 2023-11-22

**Authors:** Yuhan Yang, Zhenzi Yu, David S. Sholl

**Affiliations:** † School of Chemical & Biomolecular Engineering, Georgia Institute of Technology, Atlanta, Georgia 30332-0100, United States; ‡ Oak Ridge National Laboratory, Oak Ridge, Tennessee 37831, United States; § School of Chemical Engineering and Technology, Hainan University, Haikou 570228, China

## Abstract

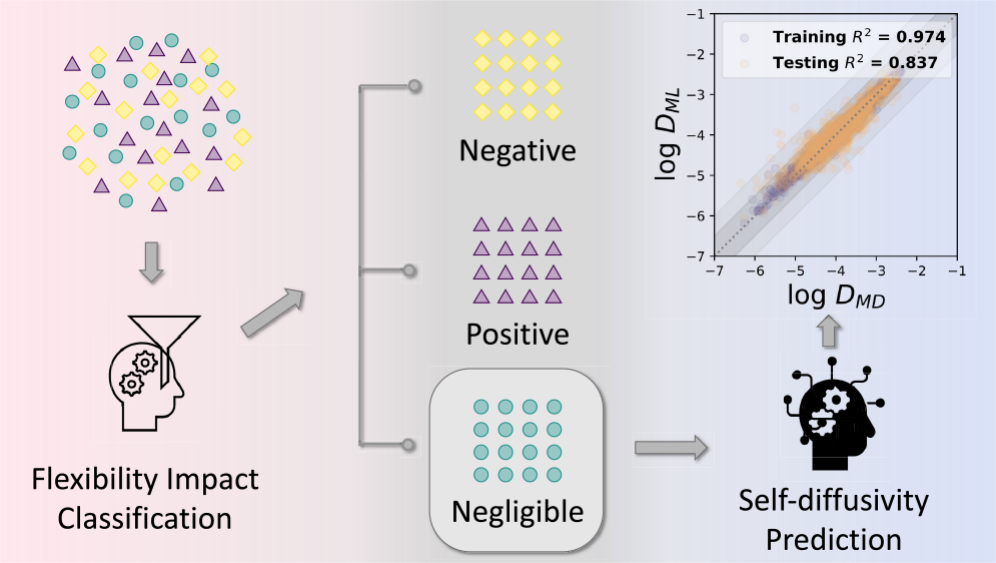

Molecular diffusion in MOFs plays an important role in determining whether equilibrium can be reached in adsorption-based chemical separations and is a key driving force in membrane-based separations. Molecular dynamics (MD) simulations have shown that in some cases inclusion of framework flexibility in MOF changes predicted molecular diffusivities by orders of magnitude relative to more efficient MD simulations using rigid structures. Despite this, all previous efforts to predict molecular diffusion in MOFs in a high-throughput way have relied on MD data from rigid structures. We use a diverse data set of MD simulations in flexible and rigid MOFs to develop a classification model that reliably predicts whether framework flexibility has a strong impact on molecular diffusion in a given MOF/molecule pair. We then combine this approach with previous high-throughput MD simulations to develop a reliable model that efficiently predicts molecular diffusivities in cases in which framework flexibility can be neglected. The use of this approach is illustrated by making predictions of molecular diffusivities in ∼70,000 MOF/molecule pairs for molecules relevant to gas separations.

## 1 Introduction

Compared with the traditional heat-driven separation technologies like distillation, evaporation, and drying, diffusion-based separations have the potential to be more energy efficient and therefore lower emissions and pollution.^1−4^ In this approach, mixtures of molecules are separated by porous materials based on their geometric or chemical properties in a diffusion process. Finding materials that have the “right” pores is critical for diffusion-based separation to succeed. Metal–organic frameworks (MOFs) can play a useful role in this field because their monodisperse pore architectures offer the potential to precisely control molecular diffusion.^5,6^

Because tens of thousands of distinct MOF materials exist,^7,8^ systematic experimental screening of potential materials for membrane- or kinetic-based separations is infeasible. Computational methods to accurately predict molecular diffusivities in MOFs now exist but are computationally intensive,^9−11^ making them impractical for large libraries of molecules and MOFs. It is natural to consider machine learning (ML) as a means to help material screening in this context. Keskin and co-workers studied the separation properties of MOF-based membranes on small molecules such as H_2_, He, CH_4_, N_2_, and O_2_.^12,13^ In their work, ML models were trained with data from molecular simulations to evaluate gas permeabilities and membrane selectivities of 5249 MOFs and 31,494 MOF/polymer mixed matrix membranes for six different gas separations: He/H_2_, He/N_2_, He/CH_4_, H_2_/N_2_, H_2_/CH_4_, and N_2_/CH_4_.^13^ Bai et al. used several ML methods to explore structure–performance relations of MOF membranes for hydrogen separation and predicted the performance of multiple top-performing MOF membranes surpassing experimental results.^14^ Zhou et al.^15^ assessed the performance of MOFs from the CoRE MOF 2019 database on D_2_/H_2_ separation using physics-based modeling. With ML, they identified MOFs whose predicted selectivity was higher than those previously reported nanoporous materials by one order of magnitude.

An important limitation of all previous high-throughput approaches to predicting diffusion in MOFs is that they are based on molecular simulations with rigid frameworks^12,13,15−18^ Simulations of this kind can be far more computationally efficient than simulations that allow flexibility of MOF degrees of freedom. There are many situations, however, in which neglecting framework flexibility leads to highly inaccurate predictions of diffusivities.^9,19−25^ A detailed discussion of this issue can be found in our previous work,^9^ and the molecular simulations described in that work are the most diverse collection of molecular diffusivities in MOFs available to date that accounts for MOF framework flexibility. Because the impact of framework flexibility on molecular diffusion in MOFs is decisive in some but not all cases, it would be very helpful to know in advance when this effect is negligible so that computationally efficient simulations based on a rigid framework could be used reliably. In this work, we further expanded the above data set to suit the needs of ML and developed a classification model to efficiently identify these situations.

Many papers applying ML to properties of MOFs and related materials have relied on macroscopic geometrical features such as the pore-limiting diameter (PLD),^26^ largest cavity diameter (LCD), surface area, pore volume, and void fraction.^27,28^ Qiao et al.^29^ calculated the relative importance of features and found that the PLD was the most important feature for MOF membrane permeability and permselectivity. A similar conclusion was given in the study of MOF membranes for D_2_/H_2_ separation by Zhou et al.^15^ These parameters are readily available in the CoRE MOF database.^30^ In this paper, we explore several additional kinds of descriptors, including energy-based and atomic property-weighted radial distribution function (AP-RDF) features to capture features of the potential energy surface of molecules in a diverse range of MOFs^31,32^ and cheminformatics features of adsorbate molecules.

In this paper, two ML models that aim to predict the molecular diffusion behaviors of various molecules in a diverse set of MOFs were developed. First, a classification model was trained to qualitatively categorize the impact of framework flexibility on molecular diffusion. This model shows that only a fraction of the data available from previous high-throughput simulations of molecular diffusion in MOFs using rigid materials is reliable. Subsequently, a regression model to quantitatively predict diffusion coefficients was developed based on an extensive collection of molecular simulation data for cases in which the influence of framework flexibility is small. The applicability of this approach is demonstrated by predicting the self-diffusivities for a wide range of industrially relevant gas molecules in the library of MOFs from the CoRE MOF database.^30^ This is a useful step toward identifying MOFs that can be used to make high-performance membranes for diffusion-based chemical separations. We have not attempted in this article to resolve the challenging task of quantitatively predicting molecular diffusivities in cases where framework flexibility has a strong effect, even though our results make predictions for which MOF/molecule pairs fall into this category. Individual examples with this property can, of course, be treated with MD simulations, but the enormous number of MOF/molecule pairs that exist makes this direct approach challenging. We hope that our work provides motivation for future efforts to expand the number of examples for which detailed MD data are available and can be used to focus the scope of future simulations of this kind.

## 2 Methods

### 2.1 Diffusion Data

Throughout this paper, the diffusion coefficients that are considered are self-diffusion coefficients at dilute loadings. At these loadings, the self-diffusion, corrected diffusion, and Fickian diffusion coefficients are exactly equal.^33^ The connections between diffusion in the dilute limit and the loading dependence of these three diffusion coefficients in nanoporous materials have been extensively explored.^34−36^

**Figure 1:**
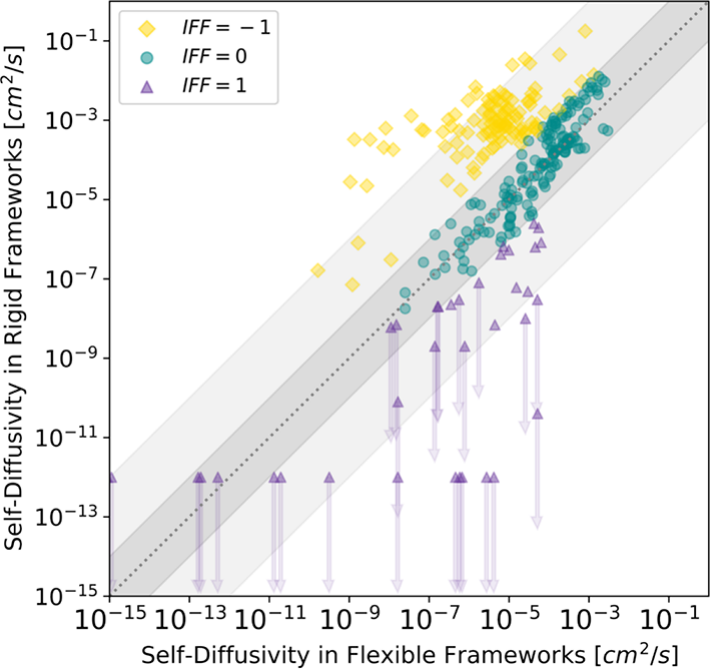
Parity plot of self-diffusivities in rigid and flexible frameworks from the Yang data set. The impact of framework flexibility (IFF) is shown by the symbol color and shape. Downward arrows indicate cases in which the MD simulation for rigid frameworks is poorly converged and provides only an upper bound. The dashed line indicates where the self-diffusivity in a rigid framework equals that in a flexible framework. The dark shading (light shading) indicates where the deviation between the flexible and rigid results is less than one order of magnitude (three orders of magnitude).

Diffusion data from rigid and flexible MOFs was available for a diverse set of 12 molecules in 17 MOFs from our previous work.^9^ We expanded this data set using another 10 MOFs that were selected following the same scheme from the CoRE MOF 2019 database. Briefly, the original 17 MOFs and structures that are 2D, publicly unavailable, or disordered materials were excluded. Then, structures with moderate PLDs (2–8 Å) and considerably larger cavities (LCD–PLD > 4 Å) were subsampled by the nearest-neighbor subsampling (NNS) algorithm to maintain diversity.^37^ In the remaining collection, structures were screened by optimization of stability and manual examination. The self-diffusion coefficients of 12 molecules in each of these 10 MOFs were calculated for rigid and flexible frameworks at infinite dilution and 308 K using the same methods as our earlier work.^9^ The data from these combined sets of simulations are shown in Figure 1 and also available in GitHub and will be referred as the Yang data set below. Figure 1 includes many examples in which the diffusivities predicted by simulations with rigid and flexible MOFs differ by multiple orders of magnitude.

To further enrich the data set for the regression model, self-diffusion coefficients of H_2_, He, CH_4_ N_2_, and O_2_ in ∼5000 selected MOFs were adapted from the published work of Keskin and co-workers.^12,13^ This data set, containing about 19,000 diffusion coefficients calculated using equilibrium MD with NVT ensemble in rigid frameworks at low but finite loadings and at 298 K, is also in GitHub from the original authors and will be referred to below as the Keskin data set. The force field used for MOFs in the Keskin data set is UFF, which is equivalent to the UFF4MOF used in the Yang data set for rigid frameworks. However, the force fields for adsorbate molecules in the two data sets are partially different (see Table S1), the loading in the Keskin data set was determined at 1 bar, and the temperatures used in the Yang data set are 10 K higher, meaning that the resulting self-diffusion coefficients are not precisely consistent. Considering these inconsistencies, the Yang and Keskin data sets were not combined and molecular force field parameters, temperature, and pressure were not included in the ML feature sets described in Section 2.2. The loading dependence of self-diffusion in the low loading regime is expected to be small in MOFs, so the small differences in loading and temperature between the two molecular simulation data sets are not expected to lead to significant variations in diffusion coefficients.

### 2.2 Features

Several features associated with the structure of MOFs were used, specifically the LCD, PLD, and specific volume (1/ρ), which are available in the CoRE MOF 2019 database.^30^ In addition, the AP-RDF features introduced by Fernandez et al.,^38^ which characterize the weighted likelihood of finding atom pairs in the MOF structure at a specified distance *R*, were used. As in the work by Fernandez et al., we consider features of this form only for atoms in the MOF frameworks. The AP-RDF function is defined by
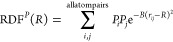
1where *r*_*ij*_ is the atom–atom distance, *P_i_* and *P_j_* are atomic properties, and *B* is a unitless smoothing parameter. We selected four atomic properties to incorporate chemical features of MOFs: electronegativity,^39^ polarizability,^40^ van der Waals volume,^41^ and atomic mass.^39^ Except for the atomic mass, the other three categories describe the MOF structures in terms of energy. *P* values for four weighting schemes are adapted from the work of Harris and Yung.^42^ A value of 10 was chosen for *B* after examining the feature profiles of various possible *B* values (Figure S1). Values of *R* ranging from 2 to 20 Å with an increment of 1 Å were used. This generated 19 features for each weighting scheme, giving a total of 76 AP-RDF features. Because the number of atoms in each MOF unit cell varies from dozens to thousands, each feature was normalized by the number of non-hydrogen atoms in the unit cell. Values of the AP-RDF features are tabulated on GitHub.

For molecules, mass, polarity, and flexibility were chosen as features, in which the polarity and flexibility are binary variables. Nonpolar molecules are labeled as “0″, while dipolar and quadrupolar molecules were labeled as “1” without distinction. The flexibility of molecules was defined based on their force field (FF) model, where “1” and “0” correspond to FF models with and without internal freedom. In addition, a number of cheminformatics features were included to describe the molecules in terms of shape, branching, connectivity, lipophilicity, etc. The BalabanJ,^43^ BertzCT,^44^ Ipc,^45^ HallKierAlpha (Chi and Kappa),^46^ MolLogP,^47^ and TPSA^48^ values were calculated based on the topological fingerprints using the RDKit package.^49^ The range of each feature was normalized to [0, 1] with min–max scaling, where 0 and 1 correspond to the minimum and maximum values of the feature.

### 2.3 ML Models

Since the Yang data set contains simulated self-diffusivities in both rigid and flexible frameworks, a classification model was trained with this data set to predict the impact of framework flexibility (IFF) on molecular diffusion in a MOF/molecule pair. A random 70/30 training/testing split was performed within the data set. To avoid overfitting the data, the correlation matrix and linear discriminant analysis (LDA)^50,51^ was used to reduce the feature dimensions. Three algorithms, namely, K-nearest neighbor (KNN), random forest (RF), and naïve Bayesian (NB), were compared by accuracy and precision.

The resulting classification model was then applied to the Keskin data set, which contains data from only simulations in rigid structures, to identify cases where the rigid framework assumption is appropriate. Data from the collection of MOF/molecule pairs for which the assumption of a rigid framework had been validated in this way were employed to train a regression model to quantitatively predict the self-diffusion coefficients that would be obtained from simulations using rigid MOF structures. Three algorithms, namely, gradient boosting regression (GBR), random forest regression (RFR), and kernel ridge regression (KRR), were compared by the coefficient of determination (*R*^2^) and mean absolute error (MAE). All ML models were developed by using the scikit-learn package.^52^

## 3 Results and Discussion

### 3.1 Classification Model Predicting the Impact of Framework Flexibility on Molecular Diffusion in MOFs

As noted above, although there are many cases in which framework flexibility plays a crucial role in molecular diffusion in MOFs, there are situations of practical interest in which more computationally efficient simulations with rigid frameworks yield accurate results. We have previously shown that targeted MD simulations with flexible frameworks, for example, of the window size distribution when a diffusing molecule is constrained to lie in the window can be used to obtain information that can help account for the importance of framework flexibility on molecular diffusion.^9^ Although this kind of information can be generated for specific materials of interest, it is not readily available for large collections of materials. Our aim of this section is therefore to develop a classification model that predicts whether inclusion of framework flexibility effects is vital for a given MOF/molecule pair without requiring inputs that can only be generated from MD simulations.

Since the Yang data set calculated the self-diffusivities for both rigid and flexible frameworks, it was used to develop our classification models. For each MOF/molecule pair in this data set, the impact of framework flexibility (IFF) was labeled based on the difference between the self-diffusivities in rigid and flexible frameworks. There are instances in the data set in which the MD simulation for rigid frameworks was poorly converged because insufficient displacements were observed during the simulation time scale.^9^ The IFF of these MOF/molecule pairs was labeled as “1”. Cases where the self-diffusivity in the rigid framework was more than one order of magnitude higher/lower than the self-diffusivity in the flexible framework were labeled as “–1”/“+1” and denoted as the “negative”/“positive” class. For the remaining MOF/molecule pairs, cases where the difference in predicted diffusivity between the flexible and rigid frameworks was less than one order of magnitude were labeled as “0” and included in the “negligible” class.

**Figure 2:**
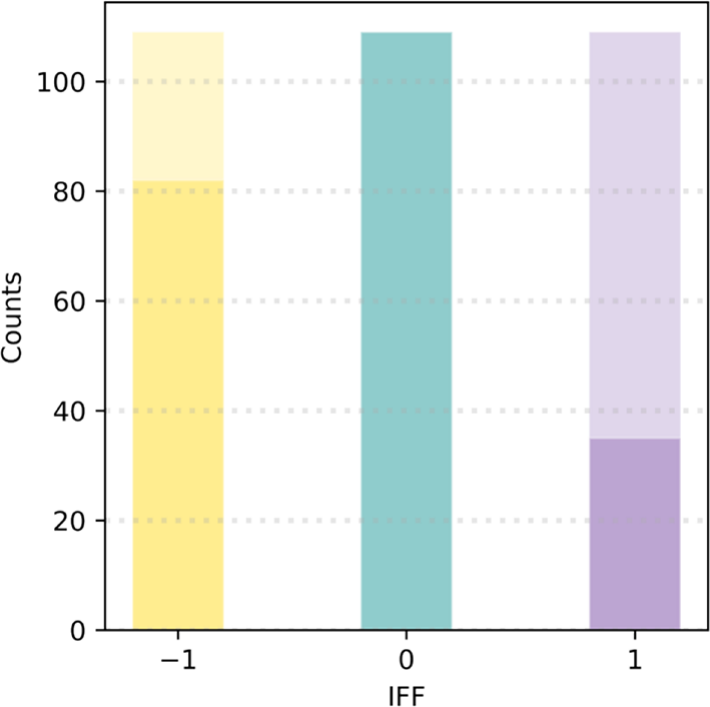
Histograms of distributions of the target variables (IFF) in the training set. Dark/light bars represent the number of observations before/after random oversampling.

A random 70/30 training/testing split was performed within the data set. The distributions of the IFF labels of the training set are shown as the dark bars in Figure 2, which show that the class distribution is unequal. Imbalanced data can cause bias toward the majority (i.e., “0”) class in the fitting process. To mitigate this effect, we employed a random oversampling method^53^ to supplement the “–1” and “+1” classes and balance the training set, as shown by the light bars in Figure 2. The final training/testing ratio is 77/23.

**Figure 3:**
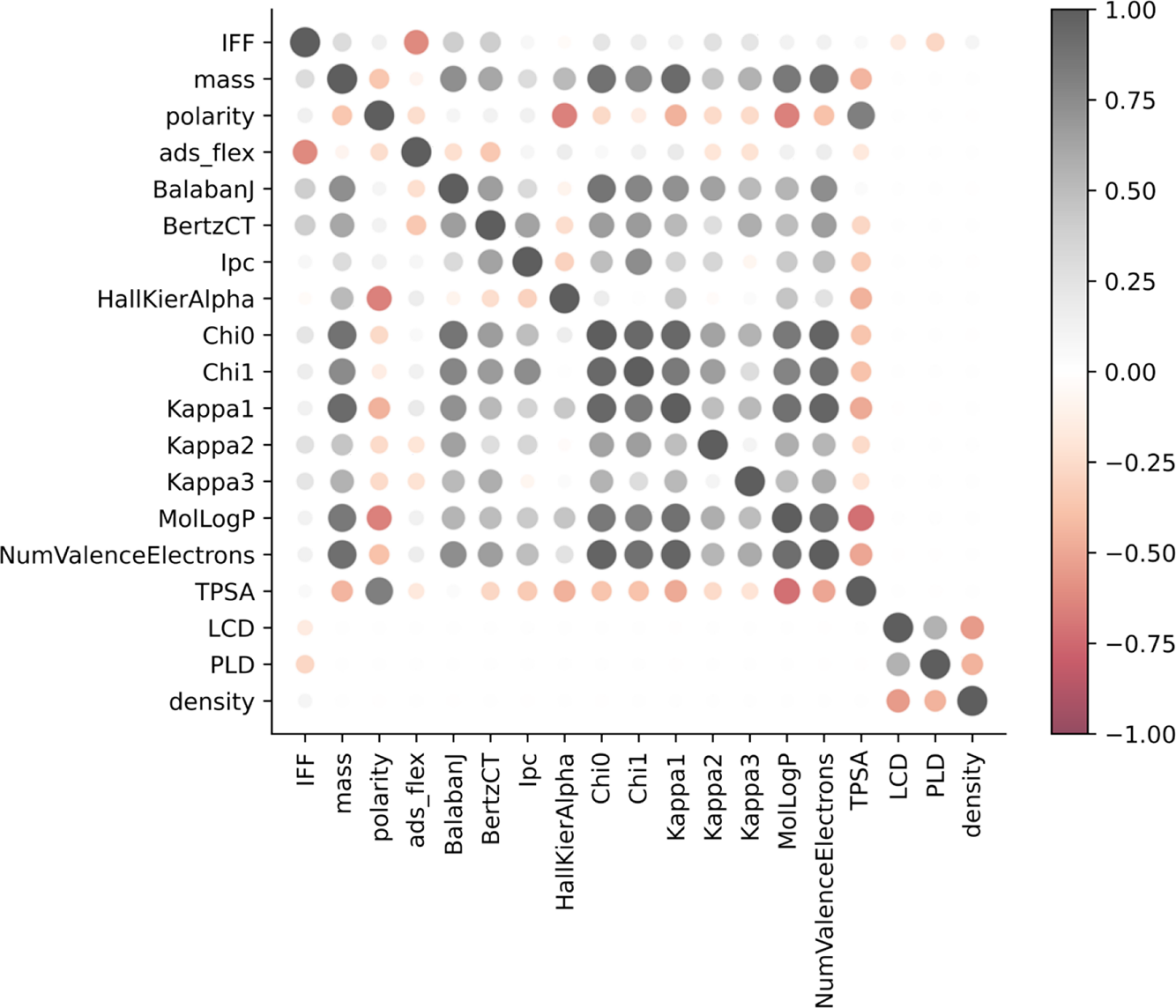
Pearson’s correlation matrix of the target variable (IFF) and the 18 basic MOF and molecule features. Value (absolute value) of each pair is shown as color (size).

The current feature set contains 18 basic features (3 MOF features and 15 molecular features) and 76 AP-RDF MOF features, which is too large for application to the available data set. To explore possible correlations among these features, the Pearson’s correlation matrix of the target variable (IFF) and the basic features is shown in Figure 3. No significant correlation between the target variable and these basic features was observed, except for the flexibility of the diffusing molecule. Consistent with the observations in our previous work,^9^ there is a strong positive correlation between a molecule being flexible and the flexibility of MOF framework strongly influencing diffusion. Among the molecular features Chi0, Chi1, Kappa1, and NumValenceElectrons are highly correlated with each other. The correlations among the AP-RDF features for the same data set are shown separately in Figure S2. As might be expected from the definition of the AP-RDF features, strong correlations exist between some of these features, indicating that a feature reduction process is appropriate. Based on this analysis, we kept one feature of each highly correlated group of features. This approach eliminated the Chi0, Kappa, and NumValenceElectrons molecular features and the polarizability, van der Waals volume, and atomic mass AP-RDF features, resulting in 34 remaining features that were used for model training.

**Figure 4:**
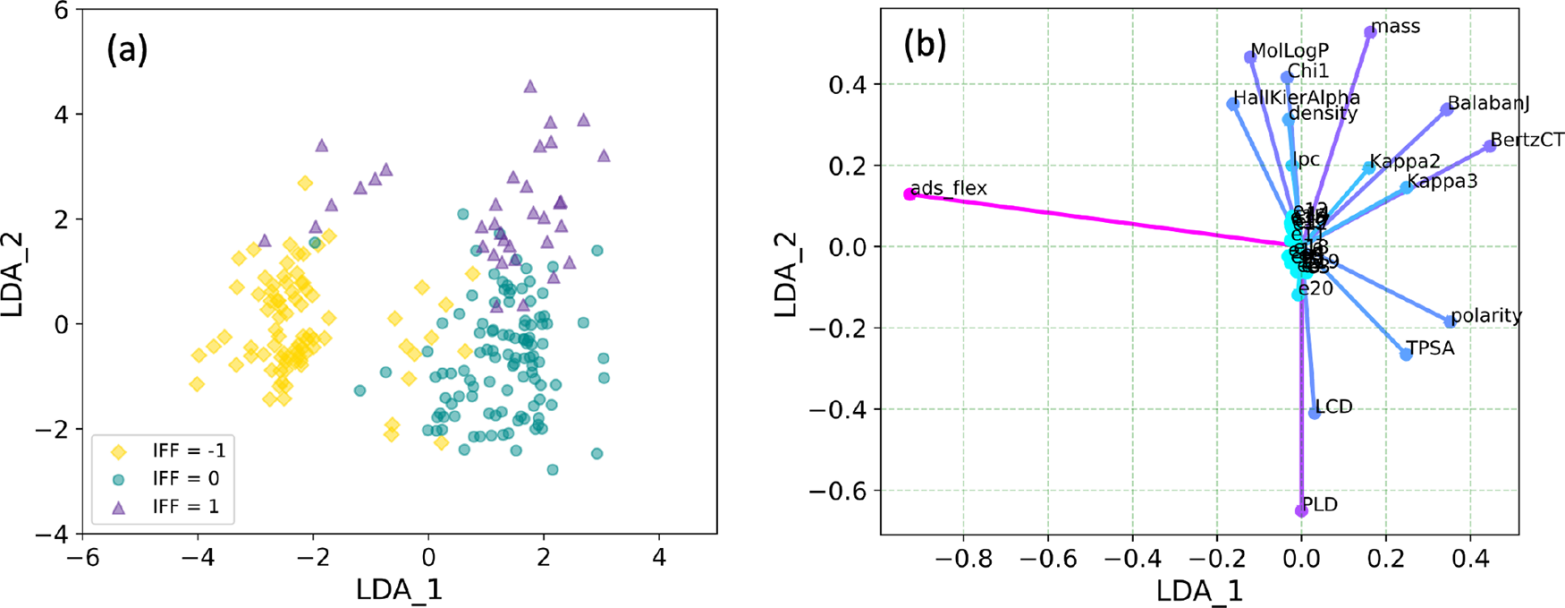
LDA projection of the Yang data set. (a) Distribution of the Yang data set in the two-dimensional LDA space, with the “negative”, “negligible”, and “positive” IFF classes shown by yellow diamonds, green circles, and purple triangles, respectively. (b) Decomposition of each feature to the LDA space; arrows are color coded according to their length for clarity.

Even after accounting for the correlation issue described above, the remaining feature set is too large given the limited size of the Yang data set. To reduce the potential for overfitting, LDA was used to further reduce the feature dimensions. LDA results in *N* – 1 linearly combined features, where *N* is the number of classes. Specifically, two LDA features were generated as inputs for the classification model. Projections of the Yang data set to the two-dimensional LDA space are shown in Figure 4. LDA can be considered a linear classifier that maximizes the distance between groups. Implementing LDA in this preprocessing step as shown in Figure 4a helps preseparate the groups and improves the performance of the classification algorithms tested below. Correlation between the original features and the LDA features shown in Figure 4b illustrates that flexibility of the molecule and PLD of the MOF contributed most toward the LDA features and, hence, might be the most influential factors in the following classification process.

Three algorithms, namely, RF, KNN, and NB, were considered for the classification model. The underlying randomness principle of RF requires that the number of features should not be too small, so RF was trained using the set of 34 features listed above while KNN and NB were applied using the two LDA features. The hyperparameters for each model were tuned using a threefold GridSearchCV. The model accuracy (eq 2) was chosen as the evaluation metric, and the hyperparameter set with the highest accuracy was selected for each model. Since predictions where impactful cases are mislabeled as negligible cases are undesirable, the precision of the models to predict cases in which the IFF is negligible (eq 3) was also considered in model selection. A trained model with low precision of negligibles was not considered. Accuracy and precision were defined as follows:
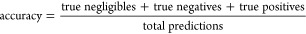
2
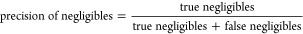
3

The accuracy and precision of negligibles are compared in Table 1. Due to the randomness of RF, 100 rounds of training were implemented with the tuned maximum depth of five and eight estimators. The distributions and means of the accuracy scores and precision scores for the training and testing sets are shown in Figure S3.

**Table 1:** Comparison of Accuracy and Precision of Negligibles of Different Algorithms

	accuracy			precision of negligibles	
	training	testing		training	testing
RF^a^	0.93	0.82		0.92	0.86
KNN	1.00	0.90		1.00	0.93
NB	0.80	0.78		0.96	0.97

a Average value of 100 training rounds.

**Figure 5:**
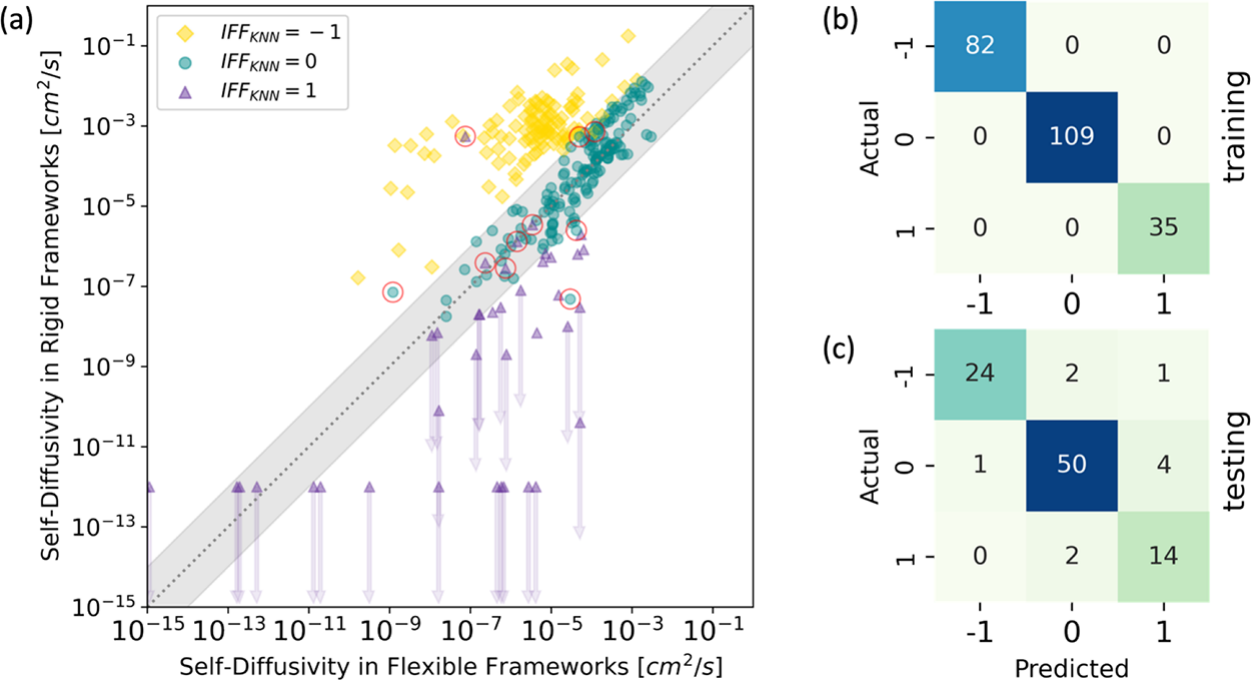
Comparison of predicted and actual IFF on molecular diffusion in the Yang data set. (a) Parity plot of self-diffusivities in rigid and flexible frameworks, with both values from molecular simulations. Symbols are color coded by the predicted label of IFF from the KNN model. The incorrect predictions are highlighted by red circles. (b, c) Confusion matrices of the training and testing data.

Generally, the testing accuracy has an upper limit at about 0.9. Figure 4 suggests two possible reasons that prevent the models from reaching higher accuracy: (1) non-Gaussian distribution of data in the training set and (2) partial overlaps around the edges of the groups. The RF model has many mislabelings of the “negligible” group and “positive” group (see Figure S4). If we focus on the precision score, the best model is NB (see Figure S5), with a model using a smoothing factor of 1.0. Based on the accuracy score, the best model is KNN. The resulting KNN model used eight neighbors and a distance-weighting strategy. The predictions of IFF from the KNN model are shown in Figure 5, which shows that only a few significant cases were mislabeled as “negligible” and the overall level of mislabeling is low. On this basis, we prefer the KNN model to the RF and NB models. This model and the implementation code are available on GitHub.

**Figure 6:**
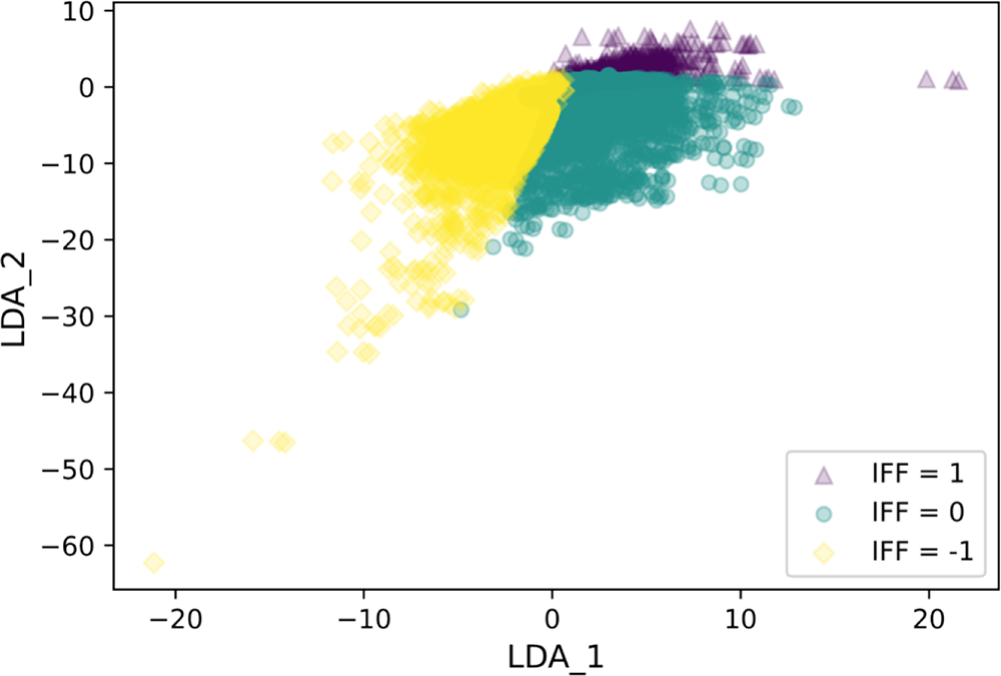
Resulting classification of the Keskin data set illustrated in the LDA space, with the same color code in Figure 4.

To illustrate the usefulness of this classification model, we applied it to the self-diffusivities from the work of Keskin and co-workers,^12,13^ which reported ∼19,000 MD self-diffusivities of H_2_, He, CH_4_, N_2_, and O_2_ from simulations with rigid MOFs. As noted above, although the Keskin data set reports diffusivities at temperatures and loadings slightly different from the MD simulations in the Yang data set, the variations associated with these differences are expected to be small. There are structures with elements for which the atomic weighting coefficients needed for the AP-RDF features are not available. After eliminating those data, we were able to classify 16,393 examples with our model. The outcomes are available in GitHub. As shown in Figure 6, 60, 34, and 6% of the data were categorized as “negligible”, “negative”, and “positive”, respectively. In other words, our analysis predicts that for 60% of the cases in the Keskin data set, it is appropriate to neglect effects associated with framework flexibility, while for the remaining cases, MD data from simulations in rigid materials are unlikely to be accurate. This is a useful addition to the Keskin data set, since previously it was not possible to assess this outcome.

**Figure 7:**
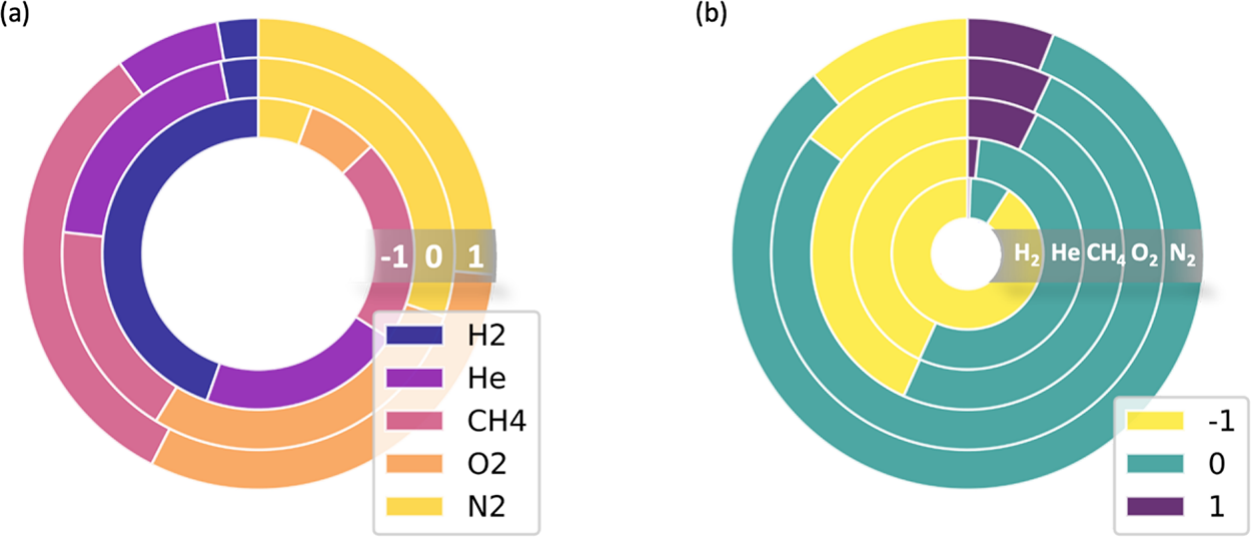
Distribution overview of (a) the fraction of cases for each molecule as a function of the predicted IFF label (−1, 0, 1) arranged from inner to outer rings, respectively, and (b) the IFF predictions for the Keskin data set for H_2_, He, CH_4_, O_2_, and N_2_ arranged from inner to outer rings, respectively.

The distribution of the predicted IFF labels in the Keskin data set is shown in Figure 7 and Table S2. Figure 7a shows the molecular distribution for each IFF class, in which the middle ring (IFF = 0) is of special interest. In Figure 7b, the fraction of cases with IFF = −1 increases as the molecule’s size decreases. An initially surprising observation is that for H_2_, ∼91% of cases were classified as situations where including framework flexibility leads to significantly slower diffusion than in rigid frameworks (IFF = −1). H_2_ was not included in the Yang data set used to train the classification model, so it is important to consider whether this prediction is reliable. In the Keskin data set H_2_, He, and CH_4_ were treated as single-site, spherical adsorbates. As shown in Figure S6, the flexibility impact indeed had the trend to negatively affect diffusivity for small spherical adsorbates and positively affect diffusivity for larger nonspherical adsorbates, which is consistent with the observations here, suggesting that our classification model has reasonable generalizability.

### 3.2 Quantitatively Predicting Self-Diffusivities for MOF/Molecule Cases Where Framework Flexibility Can Be Neglected

We now turn to the more challenging task of quantitatively predicting the self-diffusivities of molecules in MOFs. Although many physically interesting situations are cases where framework flexibility has a strong influence on diffusion, the limited amount of MD simulation data available for these cases means that applying data-driven methods to these data is not currently practical. The analysis of the Keskin data set we have just given, however, means that a large number of MOF/molecule pairs are now known for which MD data from rigid frameworks is available and the IFF has been shown to be small. Specifically, we have 9952 examples of this kind from the Keskin data set. We aimed to develop a regression model using these data to predict self-diffusivities for molecules in MOFs for which framework flexibility can be neglected. Because of the size of this data collection, we proceeded without the feature selection or projection methods that were necessary for developing the classification model in the previous section.

Three regression algorithms, namely, GBR, RFR, and KRR, were considered for this model. A random 70/30 training/testing split was performed within the 9952 data points. The self-diffusivities range over multiple orders of magnitude, which may cause numerical bias in the regression model if the values are used as the target variable directly. To reduce this effect, logarithmic values of the self-diffusivities (log *D*_self_) were used as the target variable. Hyperparameters were tuned using fivefold GridSearchCV during model development. The *R*^2^ score was chosen as the evaluation metric, and the hyperparameter set with highest *R*^2^ was selected for each model. The MAE and mean absolute percentage error (MAPE) were also calculated for the trained models. The GBR model with maximum depths of 4 and 1000 estimators outperformed the RFR and KRR models.

**Figure 8:**
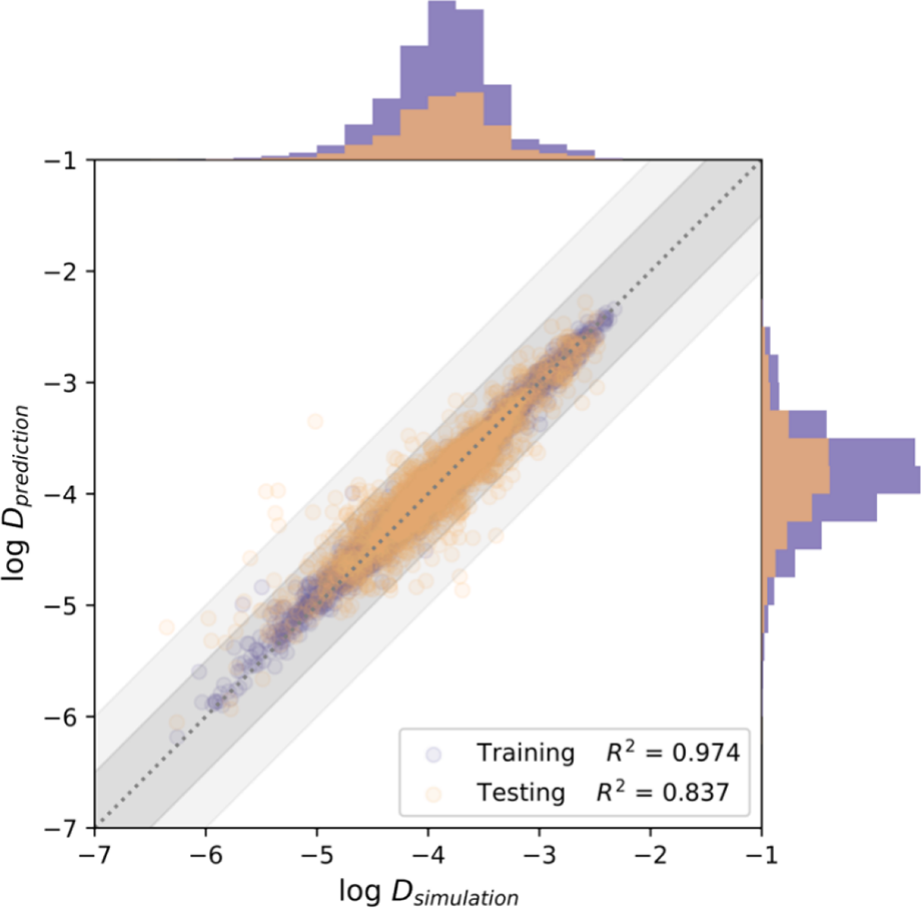
Comparison of self-diffusivities from MD simulations with rigid frameworks and model prediction for MOF/molecule pairs in the Keskin data set in which framework flexibility can be neglected. Histograms on the horizontal (vertical) axis show the distribution of the simulated (predicted) data. The light shading (dark shading) indicates where the deviation between the predicted and simulated results is less than one order of magnitude (half an order of magnitude).

The predictions of the GBR model are shown in Figure 8. The MD data includes diffusivities that vary by approximately four orders of magnitude. The data distributions for the target variable are reasonably approximated by a Gaussian distribution. Using the same data set but without excluding cases where the flexibility is impactful, Keskin and co-workers developed separate models for each individual molecule, with *R*^2^ scores of testing sets ranging between 0.65 and 0.80 and Spearman rank correlation coefficient (SPCC) values in the range of 0.75–0.89.^12,13^ Our comprehensive model has slightly higher testing *R*^2^ (0.84) and SPCC SPCC (0.92) and provides an approach that is valid for all the examined molecules, suggesting that distinguishing the flexibility impact in advance is beneficial. Although the *R*^2^ score of the training set is higher than that of the testing set, indicating the model was slightly overfitted, tuning parameters to narrow the discrepancy between training and testing sets led to strongly reduced *R*^2^ scores. The MAPE of the testing set is <5%, and the MAE of the testing sets is ∼0.12, which means the average deviation of the predicted self-diffusion coefficient from the simulated values is ∼0.12 orders of magnitude. Figure 8 shows that almost all of the predicted self-diffusivities are within half an order of magnitude from the simulated results. These deviations are less than the uncertainty associated with a typical molecular simulation, meaning that these predictions are accurate enough that they could be useful in large-scale screening of materials.

To further examine the model’s generalizability, we applied it to the Yang data set, which is independent of the regression model and has new MOFs and molecules that are outside the Keskin data set. As shown in Figure S8a, predictions for molecules common to both data sets (He, CH_4_, O_2_, and N_2_) align closely with the simulated results. There is a minor downward shift, likely attributed to the slightly lower temperature in the Keskin data set compared to the Yang data set. For the new molecules, most of the predictions of the relatively small molecules (CO_2_, H_2_O, *n*-C_4_H_10_, and *i*-C_4_H_10_) are also within one order of magnitude from the simulated results, which is satisfactory. However, for SF_6_, CCl_4_, and C_6_H_6_, which are much larger than the training molecules, the model strongly overestimated the diffusivities. These observations indicate that the current model is suitable to generalization to new MOFs but not to arbitrary new molecules. The cases where the impact of flexibility is strong were excluded in Figure S8a to keep consistency with the training procedure. If this step is not taken, the overall performance of the model is strongly degraded (Figure S8b). This illustrates again the value of distinguishing the impact of flexibility using the classification model above (or a similar approach) before predicting the self-diffusivity.

It is useful to examine which features play the strongest roles in the GBR model. Feature contributions in the model were computed using the Shapley value, which represents the contribution of each feature toward the predicted value compared to the average prediction for the data set.^54,55^ The top 20 features are summarized in Figure S9, which contains three molecule features (MolLogP, NumValenceElectrons, and mass) and 17 MOF features (PLD, density, LCD, and 14 AP-RDF features). MolLogP is the atom-based octanol–water partition coefficient developed by Wildman and Crippen.^47^ The most informative molecular and MOF features are MolLogP and PLD, respectively. No single AP-RDF feature makes a large individual contribution in Figure S9, but as might be expected these features appear to provide supplement structural information to macroscopic features like the PLD and LCD.

In applications involving equilibrium adsorption, molecular diffusion is of interest only to ensure that it is “fast enough” for equilibrium to be reached. All of the situations where our model is relevant are cases where diffusion can readily be simulated using MD, and this corresponds to situations where in experimental practice diffusion is fast enough to not cause challenges in reaching equilibrium. In other settings, notably in membrane-based separations, differences in diffusivities between different species can be exploited to achieve chemical separations.^6,56,57^ It is therefore interesting to determine whether our GBR model can accurately predict the ratios of diffusion coefficients.

**Figure 9:**
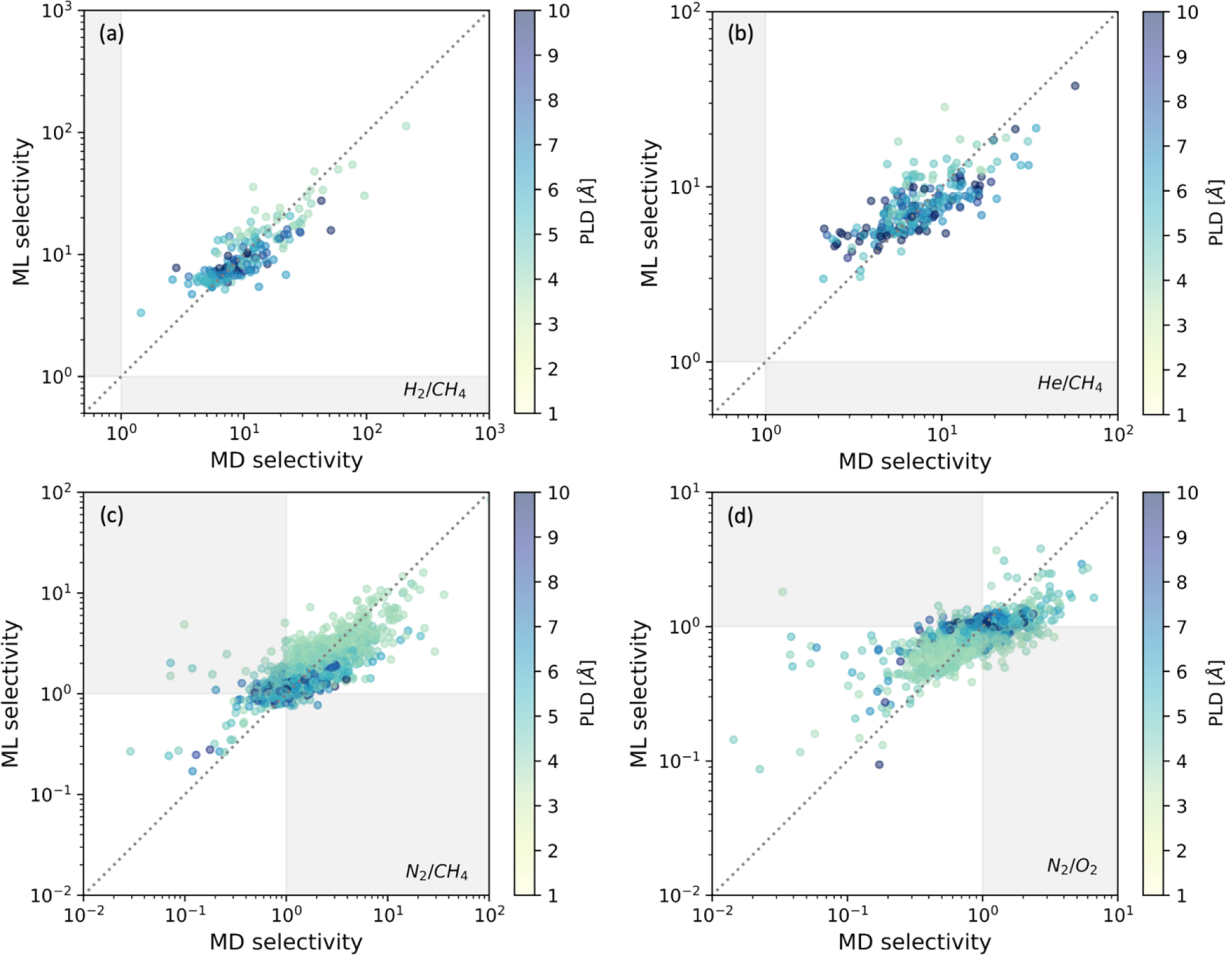
Parity plot of ideal diffusion selectivities of (a) H_2_/CH_4_, (b) He/CH_4_, (c) N_2_/CH_4_, and (d) O_2_/N_2_ calculated from MD data (horizontal axes) and ML predictions (vertical axes). Data points are color coded based on PLD of each MOF. Shadings in light gray indicate cases where the MD and ML make qualitatively different predictions for which molecules in the mixture diffuse faster.

Figure 9 compares the ideal diffusion selectivities (*D*s_1_/*D*s_2_) calculated from MD data and the ML predictions for four different pairs that are of industrial interest, H_2_/CH_4_, He/CH_4_, N_2_/CH_4_, and O_2_/N_2_. The results for the other six pairs are given in Figure S10. Qualitative agreement is achieved for most cases, and discrepancies occur more commonly when the selectivities are moderate (i.e., the ratio of diffusion coefficients ≈1). Quantitatively, the ML model tends to underestimate the diffusion selectivity for cases with higher selectivity, and this effect gets stronger as the selectivity increases. It is also observed in Figure 9 that smaller PLDs generally have higher selectivity, as might be expected.

### 3.3 An Example of High-Throughput Screening with the Classification and GBR Models

**Figure 10:**
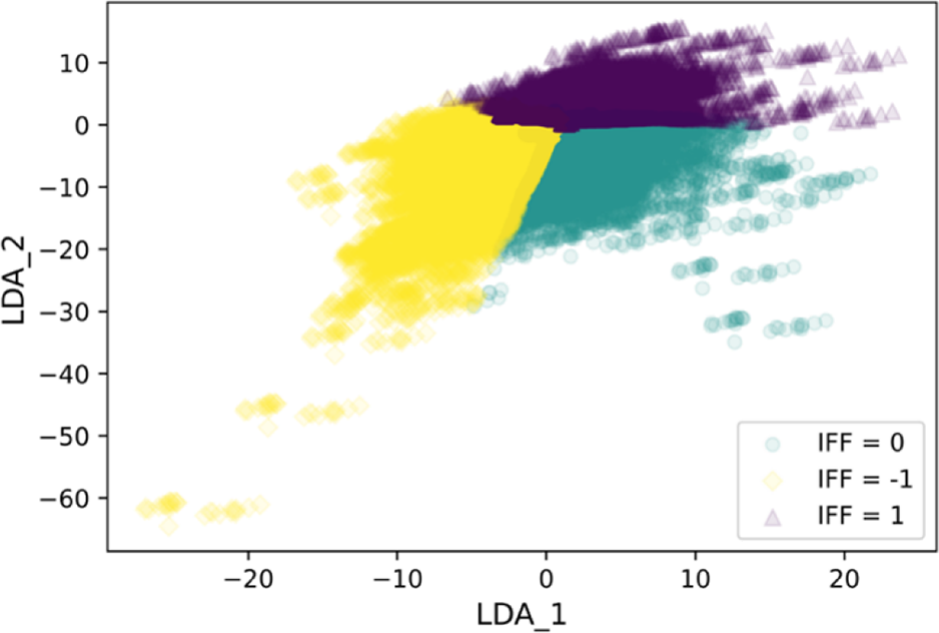
Classification of 255,204 distinct MOF/molecule pairs in the LDA space, with the same color code as in Figure 4.

To illustrate the use of our models for screening large sets of MOF–molecule pairs, we expanded the set of MOFs and molecules that we examined. For MOFs, we expanded beyond the Keskin data set to the whole CoRE MOF 2019 database. That database contains some disordered structures and structures with some elements for which the atomic weighting coefficients needed for the AP-RDF features that we used are not available. Removing those structures from consideration left a total of 9452 MOFs. To expand beyond the handful of molecules in the Keskin data set, we included the light alkane library from the work of Yu et al.^32^ As illustrated in Figure S8, it is not safe to extrapolate the GBR model to large molecules, so molecules with a mass larger than 60 in the Yu library were excluded, which resulted in a set of 27 molecules. Details of the molecular library are available in Table S3. These choices define 255,204 distinct MOF/molecule pairs. It would be extremely resource intensive to explore all of these cases with MD. We input every pair into the KNN classification model, which identified ∼70,442 (27.6%) cases where the model predicts that MD simulations based on a rigid framework assumption would be valid. The outcomes are shown in Figure 10 and are available from the example code in GitHub.

**Figure 11:**
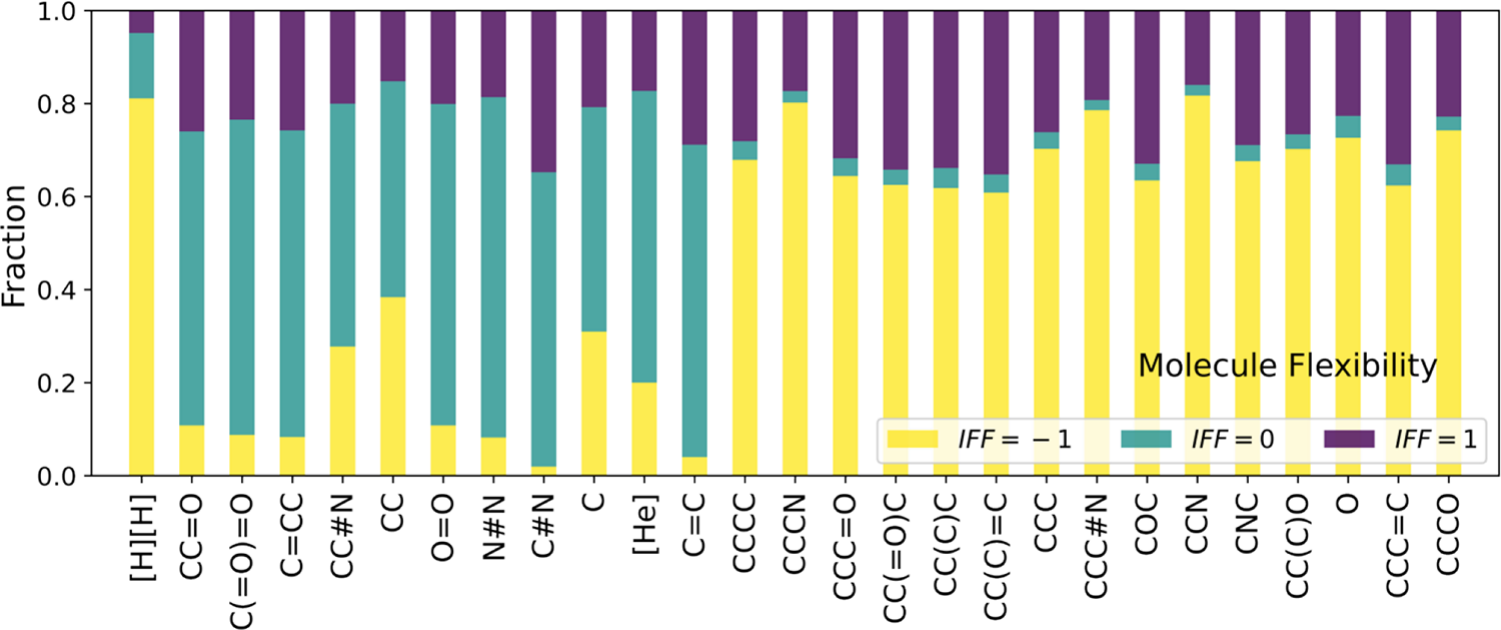
Distribution of the predicted IFF for each molecule. Molecules on the horizontal axis are sorted by the ascending value of molecule flexibility.

We studied the distribution of predicted IFF values for this collection of molecules as a function of each molecular descriptor. Figure 11 shows the descriptor that contributes most to the identification of the IFF = 0 class, with molecules ordered on the horizontal axis by ascending value of each descriptor. Consistent with the conclusion in Figure 4b and our previous work,^9^ molecular flexibility plays a critical role in determining whether framework flexibility has a strong impact on diffusion. Molecules up to and including ethene on the horizontal axis in Figure 11 are rigid molecules (within the FF our models are based upon), while those after ethene are flexible molecules. The fraction of cases with IFF = 0 is more than 40% for most rigid molecules, but this fraction drops to near zero for all flexible molecules. Although there are exceptions, the other four descriptors shown in Figure S11, mass, Kappa2&3 (shape), and Ipc (branching), also favor lower-values for cases with IFF = 0 at the lower-value region.

**Figure 12:**
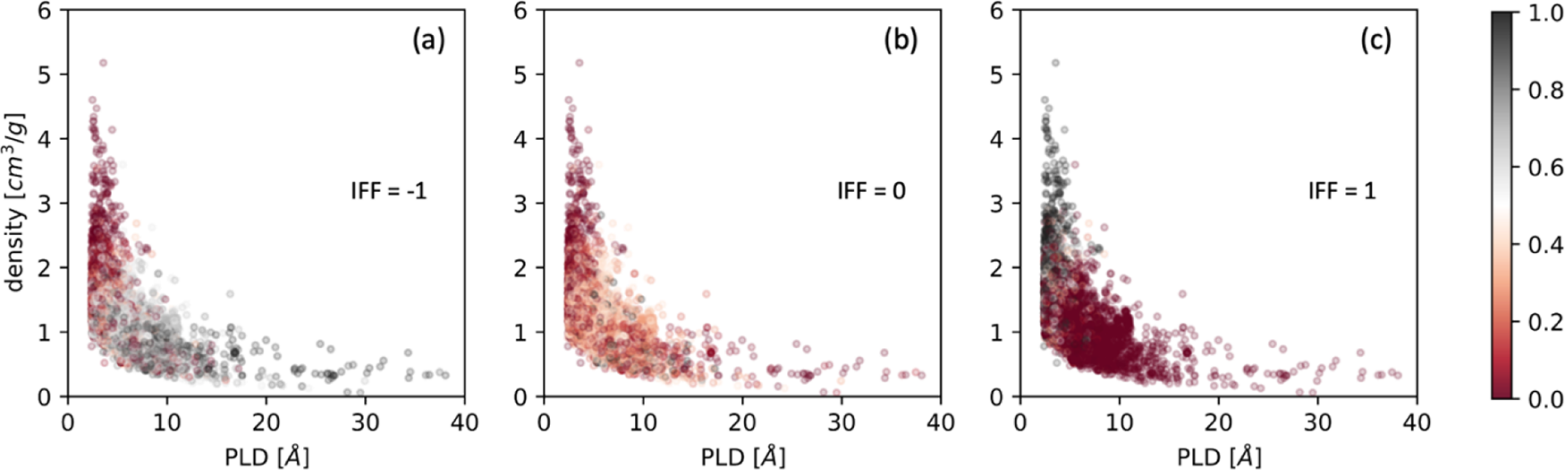
Fractions of the predicted IFF plotted against the PLD and density. The symbols are color coded by fraction of (a) IFF = −1, (b) IFF = 0, and (c) IFF = 1.

In terms of the MOF descriptors, we examined the effect of PLD and density on the fractions of the predicted IFF. Figure 12c shows that framework flexibility tends to have a positive effect for MOFs with small PLD and high density. It is perhaps more surprising that the MOFs with large PLD and low density are more likely to have a negative flexibility effect rather than a negligible effect on the diffusivities. This observation is a reminder that assuming that framework flexibility is not relevant because a MOF’s pores are large may not be wise.

**Figure 13:**
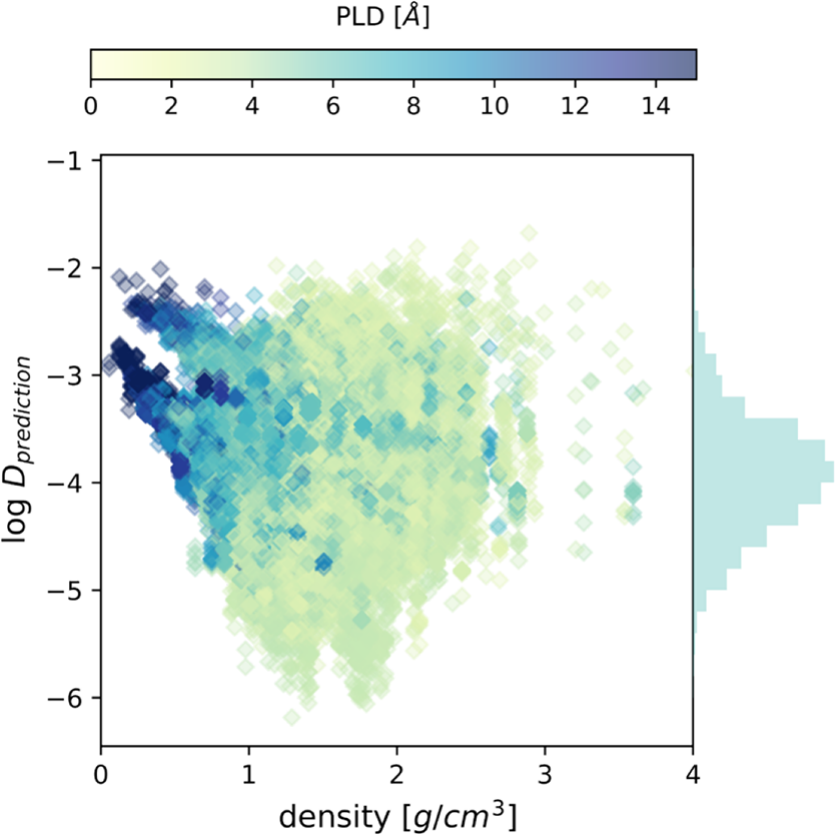
GBR model predicted self-diffusivities plotted as a function of the MOF density. Histograms on the vertical axis represent the distribution of predicted log*D*_prediction_. The symbols are color coded by PLD.

The GBR model was then applied to each of the IFF = 0 MOF/molecule pairs to predict their self-diffusivities. As shown in Figure 13, the range of self-diffusivities spans from 10^–6^ to 10^–2^ cm^2^/s, with most values lying between 10^–5^ and 10^–3^ cm^2^/s. These results provide us a rich data set to quickly search potential materials for kinetic separation of various molecules.

Of the ∼70,000 MOF/molecule pairs for which we have made quantitative predictions, 36.4% are for one of the five small molecules for which data is available in the Keskin data set (H_2_, He, CH_4_, O_2_, or N_2_). Because our GBR model was trained with data from these molecules, it is reasonable to expect that the predictions for these molecules are accurate. Diffusion selectivities of He/CH_4_ in 4505 MOFs for which framework flexibility can be neglected were calculated and are shown in Figure S12. This analysis includes 4261 more MOFs than the simulation data of Keskin and co-workers and pushes the selectivity upper bound to more than 100 from ∼40 in the Keskin collection.

**Figure 14:**
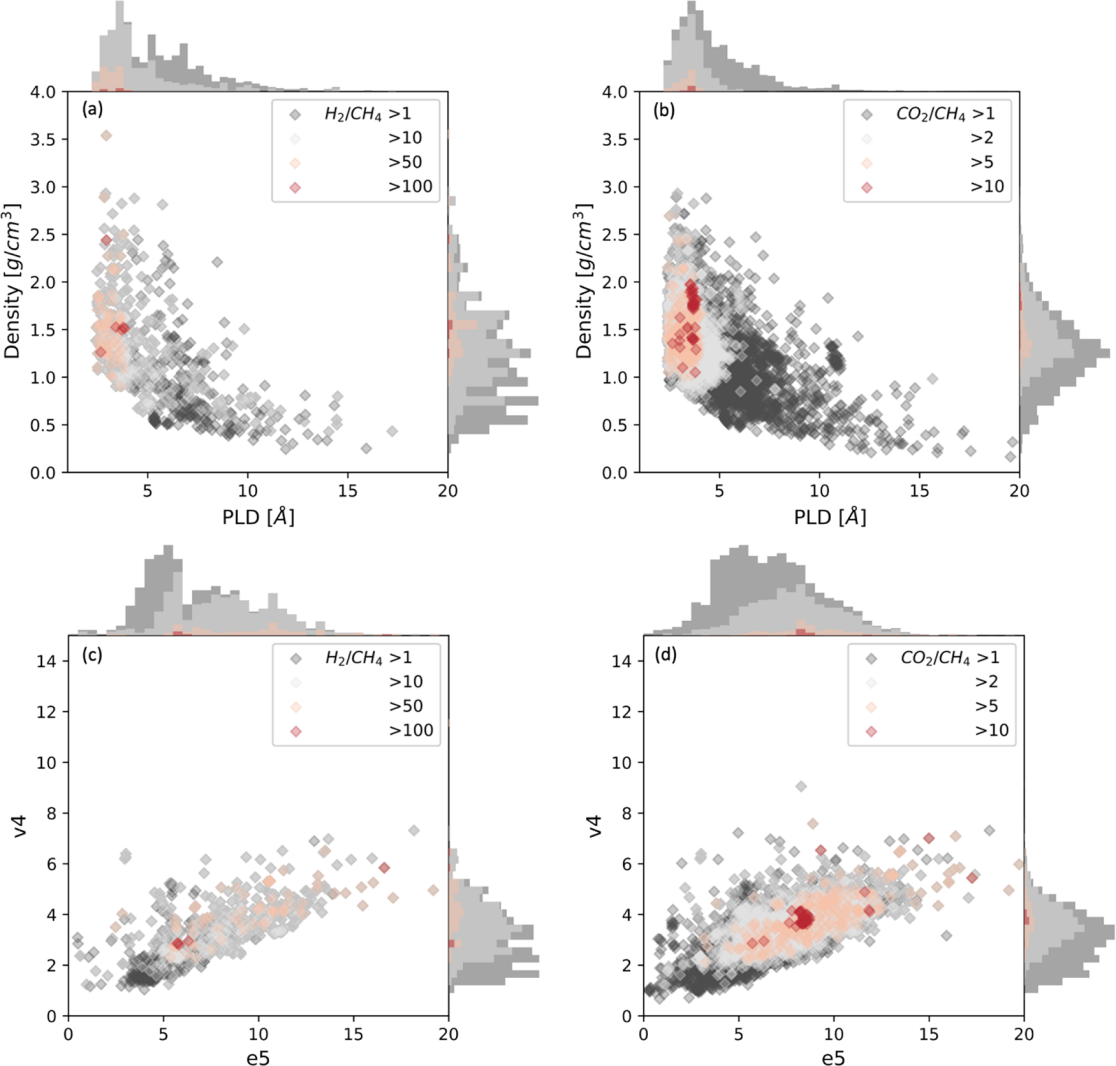
(a, b) Heatmaps of diffusion selectivity based on PLD (horizontal axis) and density (vertical axis) for H_2_/CH_4_ and CO_2_/CH_4_ diffusion selectivity, respectively; (c, d) Heatmaps of diffusion selectivity based on e5 (horizontal axis) and v4 (vertical axis) for the same two molecular pairs. In each panel, the symbols are color coded by the selectivity values; the molecule pairs and corresponding gradients are labeled at the top right corner of each subplot.

Figure 14 shows a similar analysis for the diffusion selectivity of H_2_/CH_4_ and CO_2_/CH_4_, including large numbers of materials that were not considered in the Keskin data set. The heatmap of diffusion selectivity for H_2_/CH_4_ indicates that the most selective materials have PLD ∼ 3 Å and density ∼1.5 cm^3^/g as geometric features and energetic features of e5 ∼6 and v4 ∼3, respectively. An interesting observation from Figure 14d is that compared with H_2_/CH_4_, the best region of e5 is ∼8 for CO_2_/CH_4_ while the most suitable PLDs for the two molecular pairs are similar.

## 4 Summary

In this work, a two-step ML model was developed to predict the molecular diffusion of a diverse array of molecules in large libraries of MOFs while accounting for the effects of the MOF framework flexibility on this diffusion. It is known from detailed molecular simulations that framework flexibility can have very strong effects on molecular diffusion in MOFs, but all previous attempts to describe molecular diffusion in these materials in a high-throughput way have neglected this important effect. The first step of our approach uses a classification model to predict whether diffusion data from molecular simulations that neglect framework flexibility can reliably be used in place of the more physically realistic situation where this flexibility is included. The resulting model was shown to have good accuracy and prediction in this classification task based on an extensive set of molecular simulation data from our earlier work that we supplemented in this paper. In this model, the flexibility of the molecule and the PLD of the MOF (in its rigid structure) were found to be the most significant features. A key limitation of our work is that the number of MOF/molecule examples we used for which diffusivities from fully flexible simulations were less than 350. The MOFs in these simulations were selected with methods that aimed to avoid systematic bias in terms of the material’s pore geometries (specifically, their PLD and LCD), but other factors could also be important in fully describing the rich variety and chemistry of MOFs. We hope that our work prompts additional MD simulations of diverse MOF/molecule pairs in the future to provide data that can further challenge and refine the models that we have presented.

The second step in our model makes quantitative predictions of molecular diffusivities for MOF–molecule pairs for which framework flexibility can reliably be neglected. Training this model was accomplished by combining our classification model with a large collection of MD simulations in rigid structures by Keskin and co-workers. We found that provided framework flexibility can be neglected the resulting model predicts diffusivities with accuracy comparable to conventional but much more computationally demanding MD simulations. This model shows good performance for small molecules, but its applicability to larger molecules is unclear because of the unavailability of MD data for comparisons.

To illustrate the use of our model for screening large numbers of MOF–molecule pairs, we considered the diffusion of 27 different molecules in every MOF from the CoRE MOF 2019 database. This gave ∼70,000 examples where it is reasonable to neglect framework flexibility and where quantitative estimates of molecular diffusivities have been made. This outcome greatly expands the number of MOF–molecule pairs for which reliable predictions of molecular diffusion are available. Using methane-related separations as examples, we identified the regions associated with the highest diffusion selectivities for various molecular pairs.

There are many practically interesting examples in which the framework flexibility must be included to make quantitative predictions about molecular diffusion in MOFs. In many of these cases, predictions made based on MD simulations from rigid MOFs are incorrect by multiple orders of magnitude. Although our classification model is a useful step forward in being able to identify these situations, the challenge of using ML to quantitatively predict the relevant diffusivities remains open because there are not sufficient MD data currently available to train meaningful models. MD simulations can, of course, be used to study individual cases of special interest in a detailed way, and in our view this is the only sensible approach to treating MOF/molecule pairs in which framework flexibility plays a strong role based on current information. If a diverse data set of diffusivities from MD simulations with flexible MOFs was generated in the future, methods analogous to those we have used in this paper would allow the development of robust models that could predict molecular diffusivities in all situations.

Throughout this work, we have focused on predicting molecular diffusion in MOFs that are defect-free based on molecular simulations that use generic FFs. As in all crystalline materials, real MOFs have defects in their structures.^58^ Examples are known in which defects in MOFs play a pivotal role in the characteristics of molecular diffusion.^59^ In cases of strong practical interest, using molecular simulations to probe the potential impact of defects on molecular diffusion would be wise. Methods have recently been introduced allowing creation of structural models containing point defects in large libraries of MOFs that will make simulations of this kind more feasible than in the past.^60^ Similarly, in the most interesting examples, it would be productive to assess the quality of generic FFs, a task that can be accomplished by performing systematic comparisons between molecular adsorption energies calculated with an FF and calculated with higher-level methods such as dispersion-corrected DFT.^61−64^

## Supporting Information Available

Comparison of molecular force fields between the Yang data set and Keskin data set, summary of predicted IFF labels for the Keskin data set, information on the 27 molecules, AP-RDF electronegativity feature profiles, Pearson’s correlation matrix of IFF and AP-RDF features, boxplot of accuracy and precision scores of random forest model, results of the random forest model and naïve Bayesian model, ratios of self-diffusivities between flexible framework and rigid framework of each molecule from the Yang data set, results of the random forest regression model and kernel ridge regression model, comparison of self-diffusivities from MD simulations and model prediction for the Yang data set including the flexibility impactful cases, Shapley values summary for the top 20 features in the GBR model, parity plot of ideal diffusion selectivity of gas mixtures calculated from MD and ML, distribution of the predicted IFF for the 27 molecules, and heatmaps of diffusion selectivity based on geometric features and energetic features (PDF)
LAMMPS input data files for the 10 supplementary MOFs. (ZIP)
