# Supplementary material for: Machine Learning Models for Predicting Molecular Diffusion in Metal–Organic Frameworks Accounting for the Impact of Framework Flexibility
Source: Chem Mater. 2023 Nov 22;35(23):10156–68. doi: 10.1021/acs.chemmater.3c02321 (PMC10720339; doi:10.1021/acs.chemmater.3c02321)
Supplement: Supplementary file 1 — cm3c02321_si_001.pdf [file cm3c02321_si_001.pdf]

Supporting Information for

Machine Learning Models for Predicting Molecular Diffusion in Metal-Organic Frameworks Accounting for the Impact of Framework Flexibility

Yuhan Yang[1,3], *Zhenzi Yu*[1], *David S. Sholl*[1,2] *

[1] School of Chemical & Biomolecular Engineering, Georgia Institute of Technology, Atlanta, GA 30332-0100, USA

[2] Oak Ridge National Laboratory, Oak Ridge, TN 37831, USA

[3] School of Chemical Engineering and Technology, Hainan University, Haikou, 570228, China

In addition to the tables and figures in the document below, a .zip file containing additional data is available as Supporting Information. This .zip file includes the LAMMPS input data files for the 10 supplementary MOFs.

Supporting data and resources to allow implementation of the ML models in this work are available from GitHub at https://github.com/youngyuhan/Sharing/tree/main/SI_git.

Tables

Table S1. Comparison of molecular force fields between Yang dataset and Keskin dataset.

	Yang				Keskin		
	**σ** [Å]	ε [kcal/mol]	charge	distance [Å]	**σ** [Å]	ε [kcal/mol]	charge
H_2	--	--	--	--	2.96	0.0680	0
He	2.64	0.0217	0	--	2.64	0.0217	0
CH_4	3.73	0.2940	0	--	3.73	0.2941	0
$O_{2,O}$	3.02	0.9740	-0.113	0.605 (O-com)	3.02	0.9740	-0.113
$O_{2,com}$	0	0	0.226		0	0	0.226
$N_{2,N}$	3.32	0.0724	-0.482	0.55 (N-com)	3.31	0.0761	-0.405
$N_{2,com}$	0	0	0.964		0	0	0.810

Table S2. Summary of predicted IFF labels for each molecule in Keskin dataset.

IFF	H_2	He	CH_4	N_2	O_2	Sum
-1	2158	267	1906	499	682	5512
0	202	341	2174	3653	3582	9952
1	15	10	322	262	320	929
Total	2375	618	4402	4414	4584	16393

Table S3. Information of the 27 molecules considered.

Molecule	SMILES	mass	polarity	ads_flex	BalabanJ	BertzCT	Ipc	HallKierAlpha
**helium**	[He]	4.003	0	0	0.000	0.000	0.000	-0.091
**methane**	C	16.03	0	0	0.000	0.000	0.000	0.000
**oxygen**	O=O	32	1	0	2.000	0.000	2.000	-0.400
**nitrogen**	N#N	28	1	0	3.000	4.755	2.000	-0.580
**carbon dioxide**	C(=O)=O	44	1	0	3.266	23.774	2.755	-0.620
**water**	O	18	1	1	0.000	0.000	0.000	-0.040
**butane**	CCCC	58	0	1	1.975	2.000	6.855	0.000
**isobutane**	CC(C)C	58	0	1	2.324	4.755	3.245	0.000
**hydrogen**	[H][H]	2.016	0	0	1.000	0.000	2.000	0.000
**acetaldehyde**	CC=O	44.05	1	0	2.187	10.265	2.755	-0.330
**acetone**	CC(=O)C	58.08	1	0	2.803	26.265	3.245	-0.330
**acetonitrile**	CC#N	41.05	1	0	2.476	24.265	2.755	-0.510
**dimethyl ether**	COC	46.07	1	1	1.633	2.755	2.755	-0.040
**dimethylamine**	CNC	45.08	1	1	1.633	2.755	2.755	-0.040
**ethane**	CC	30.07	0	0	1.000	0.000	2.000	0.000
**ethene**	C=C	28.05	0	0	2.000	0.000	2.000	-0.260
**ethylamine**	CCN	45.08	1	0	1.633	2.755	2.755	-0.040
**hydrogen cyanide**	C#N	27.03	1	0	3.000	6.755	2.000	-0.510
**isopropyl alchohol**	CC(C)O	60.1	1	1	2.324	8.000	3.245	-0.040
**propane**	CCC	44.1	0	1	1.633	0.000	2.755	0.000
**propene**	C=CC	42.08	0	0	2.187	7.510	2.755	-0.260
**propionitrile**	CCC#N	55.08	1	0	2.433	33.038	6.855	-0.510
**propyl alcohol**	CCCO	60.1	1	1	1.975	5.245	6.855	-0.040
**1-butene**	CCC=C	56.11	0	1	2.297	14.000	6.855	-0.260
**isobutene**	CC(C)=C	56.11	0	0	2.803	23.020	3.245	-0.260
**propionaldehyde**	CCC=O	58.08	1	1	2.297	17.245	6.855	-0.330
**propylamine**	CCCN	59.11	1	1	1.975	5.245	6.855	-0.040

Continued.

Molecule	Chi0	Chi1	Kappa1	Kappa2	Kappa3	MolLogP	NumValenceElectrons	TPSA
**helium**	0.000	0.000	0.909	-13.091	-48.091	0.000	2	0.000
**methane**	0.000	0.000	0.000	0.000	0.000	0.636	8	0.000
**oxygen**	2.000	1.000	1.600	0.600	-4.900	0.067	12	34.140
**nitrogen**	2.000	1.000	1.420	0.420	-4.304	0.030	10	47.580
**carbon dioxide**	2.707	1.414	2.380	1.380	1.380	-0.584	16	34.140
**water**	0.000	0.000	0.960	-27.040	-104.040	-0.825	8	31.500
**butane**	3.414	1.914	4.000	3.000	2.000	1.806	26	0.000
**isobutane**	3.577	1.732	4.000	1.333	0.000	1.662	26	0.000
**hydrogen**	2.000	1.000	0.000	0.000	0.000	0.246	2	0.000
**acetaldehyde**	2.707	1.414	2.670	1.670	1.670	0.205	18	17.070
**acetone**	3.577	1.732	3.670	1.045	6.884	0.595	24	17.070
**acetonitrile**	2.707	1.414	2.490	1.490	1.490	0.530	16	23.790
**dimethyl ether**	2.707	1.414	2.960	1.960	1.960	0.263	20	9.230
**dimethylamine**	2.707	1.414	2.960	1.960	1.960	-0.164	20	12.030
**ethane**	2.000	1.000	2.000	0.000	0.000	1.026	14	0.000
**ethene**	2.000	1.000	1.740	0.740	-6.106	0.802	12	0.000
**ethylamine**	2.707	1.414	2.960	1.960	1.960	-0.035	20	26.020
**hydrogen cyanide**	2.000	1.000	1.490	0.490	-4.471	0.140	10	23.790
**isopropyl alchohol**	3.577	1.732	3.960	1.298	1128.960	0.387	26	20.230
**propane**	2.707	1.414	3.000	2.000	0.000	1.416	20	0.000
**propene**	2.707	1.414	2.740	1.740	1.740	1.192	18	0.000
**propionitrile**	3.414	1.914	3.490	2.490	1.490	0.920	22	23.790
**propyl alcohol**	3.414	1.914	3.960	2.960	1.960	0.389	26	20.230
**1-butene**	3.414	1.914	3.740	2.740	1.740	1.582	24	0.000
**isobutene**	3.577	1.732	3.740	1.105	14.095	1.582	24	0.000
**propionaldehyde**	3.414	1.914	3.670	2.670	1.670	0.595	24	17.070
**propylamine**	3.414	1.914	3.960	2.960	1.960	0.355	26	26.020

Figures

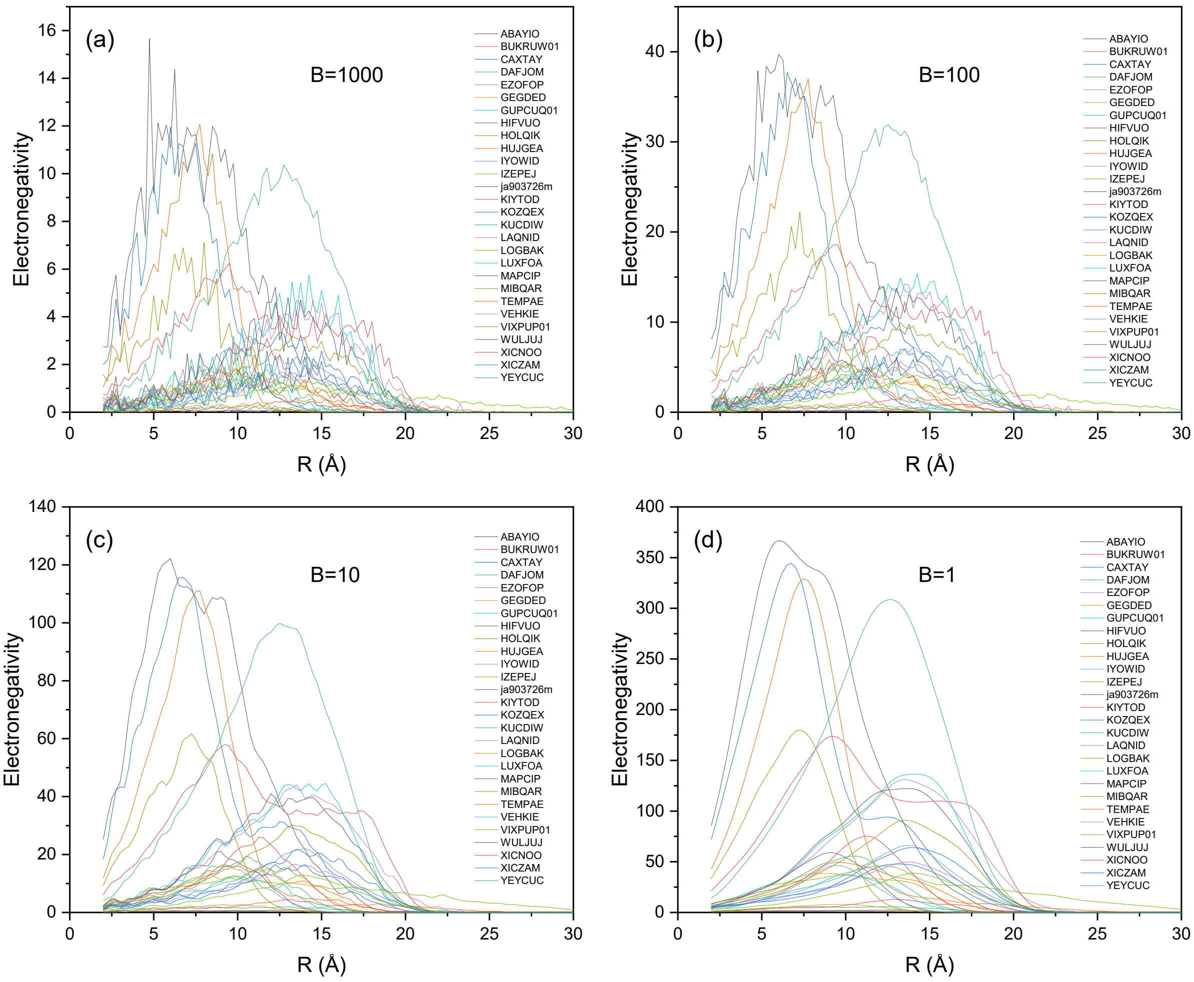

Figure S1. AP-RDF feature (electronegativity) profiles of different B values: (a) B=1000, (b) B=100, (c) B=10, (d) B=1.

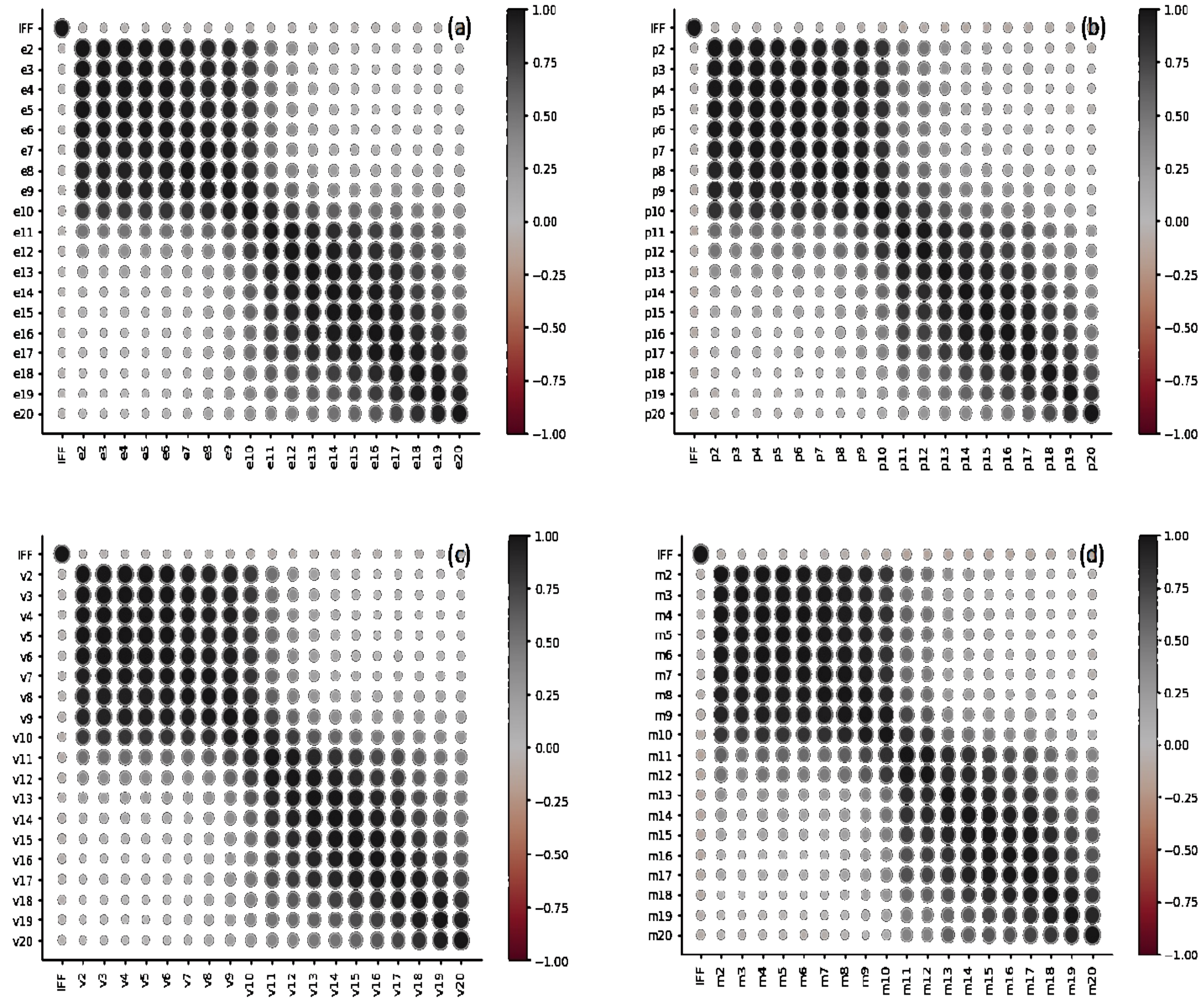

Figure S2. Pearson's correlation matrix of the target variable (IFF) and the AP-RDF features. (a) electronegativity, (b) polarizability, (c) van der Waals volume and (d) atomic mass. Value (absolute value) of each pair is shown as color (size).

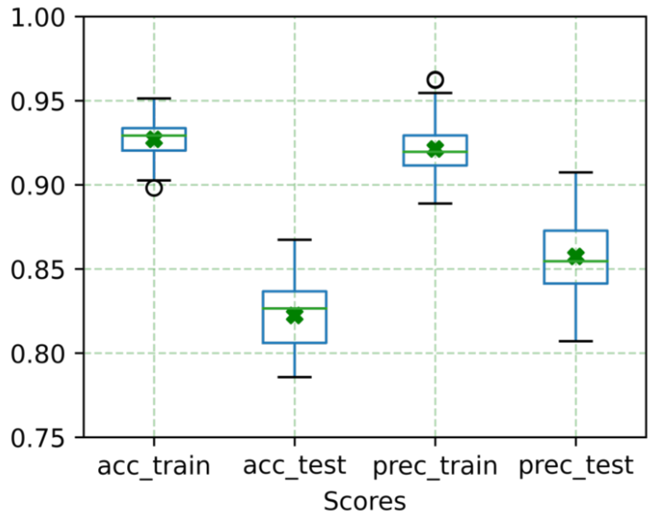

Figure S3. Boxplot of the accuracy scores and precision scores for training and testing sets in the 100 training rounds of random forest model. Green crosses are the mean of each distribution.

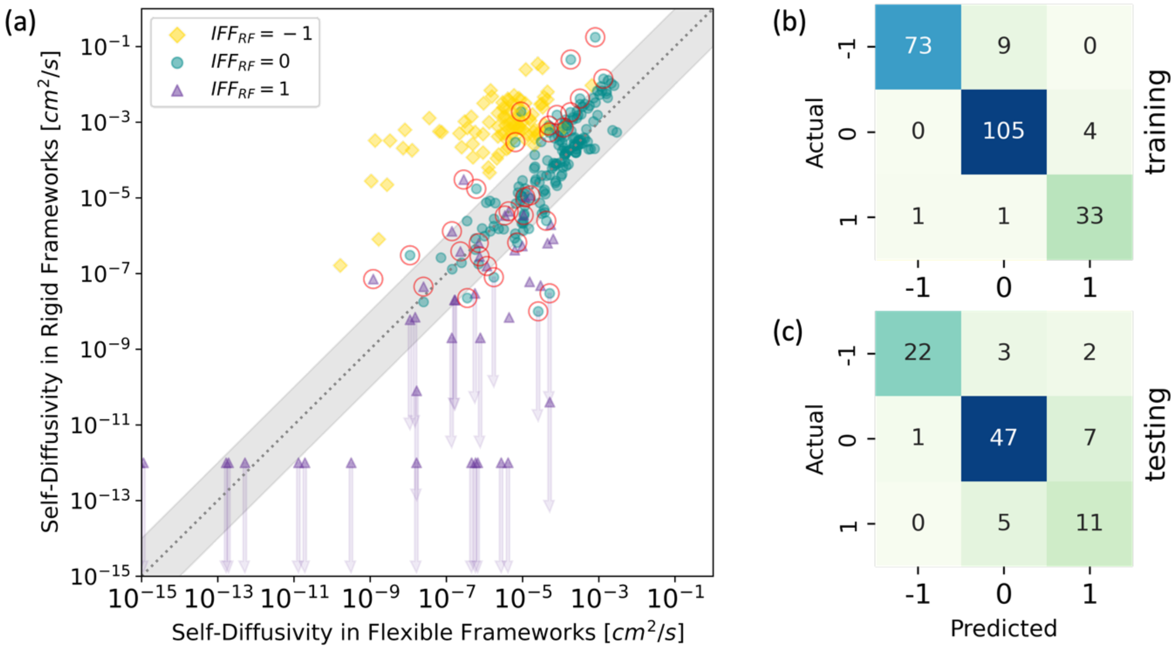

Figure S4. Comparison of predicted and actual IFF on molecular diffusion in original Yang dataset. (a) Parity plot of self-diffusivity in rigid and flexible frameworks, with both values from molecular simulations. Symbols are color coded by predicted IFF from the RF model. Incorrect predictions are highlighted by red circles. (b) and (c) Confusion matrices of the training and testing data.

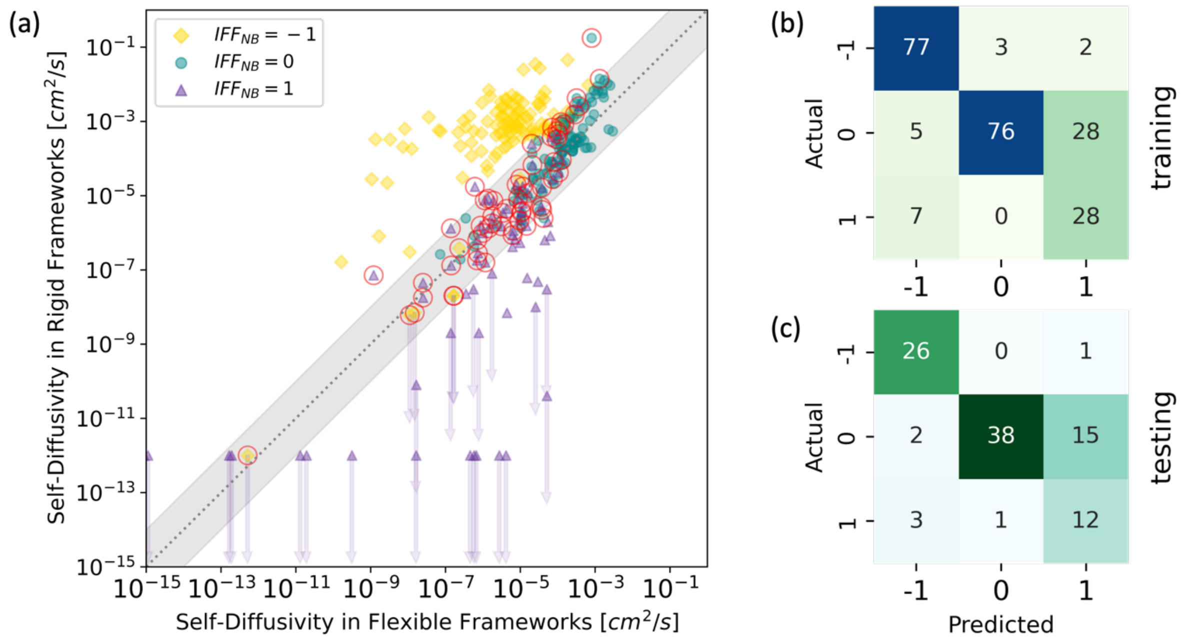

Figure S5. Comparison of predicted and actual IFF on molecular diffusion in original Yang dataset. (a) Parity plot of self-diffusivity in rigid and flexible frameworks, with both values from molecular simulations. Symbols are color coded by predicted IFF from the NB model. Incorrect predictions are highlighted by red circles. (b) and (c) Confusion matrices of the training and testing data.

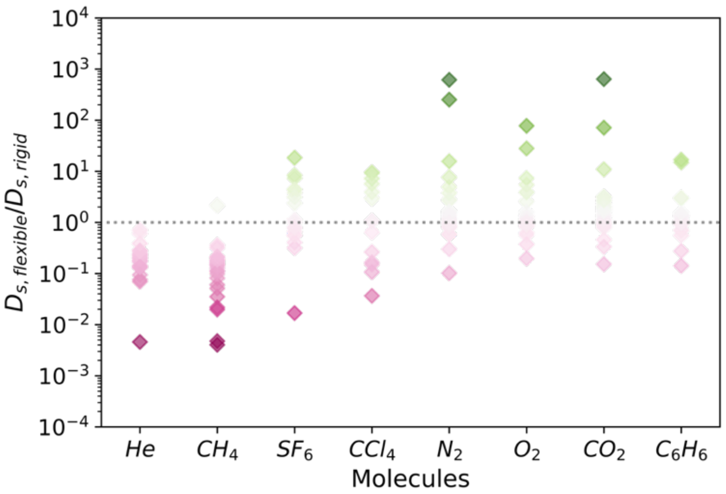

Figure S6. Ratios of self-diffusivities between flexible framework and rigid framework of each molecule from the Yang dataset. Cases containing poorly converged rigid self-diffusivities or flexible molecules were eliminated. Symbols are color coded by the value of ratios.

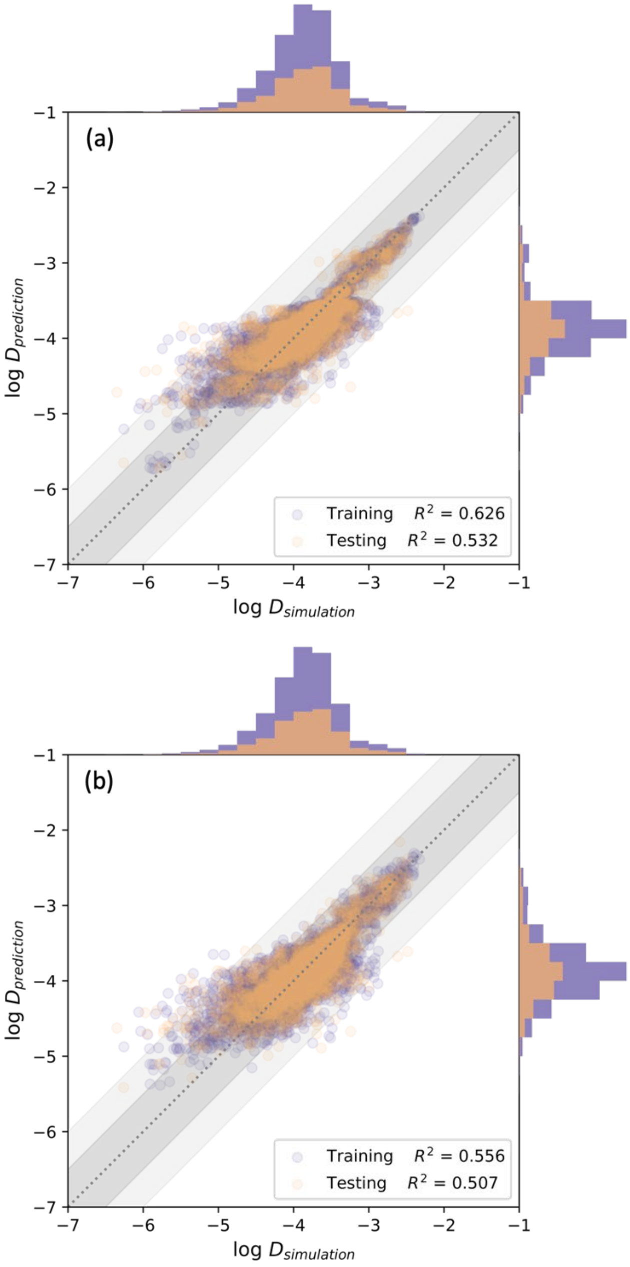

Figure S7. Comparison of self-diffusivities of the "negligible" class between simulation and prediction from (a) RFR model and (b) KRR model. Histograms on the x (y) axis is the distribution of simulated (predicted) data. The light shading (dark shading) indicates where the deviation between the predicted and simulated results are less than one order of magnitude (half an order of magnitude).

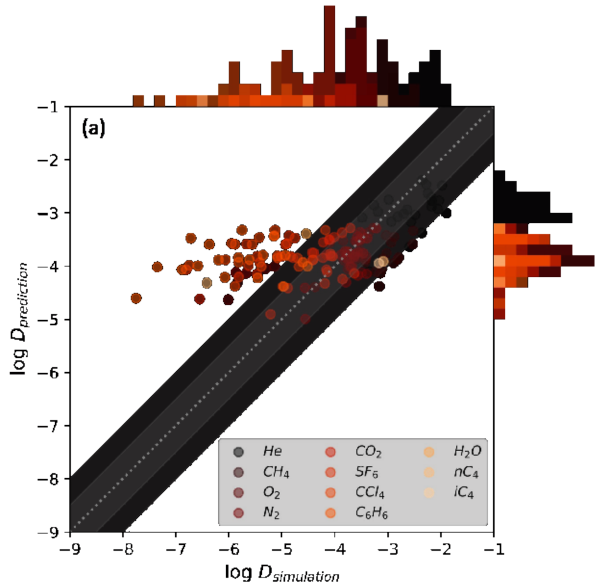

Figure S8. Comparison of self-diffusivities from MD simulations with rigid frameworks and model prediction for MOF/molecule pairs in the Yang dataset (a) in which framework flexibility can be neglected, (b) including cases where the flexibility is impactful. Histograms on the horizontal (vertical) axis is the distribution of simulated (predicted) data. The light shading (dark shading) indicates where the deviation between the predicted and simulated results are less than one order of magnitude (half an order of magnitude).

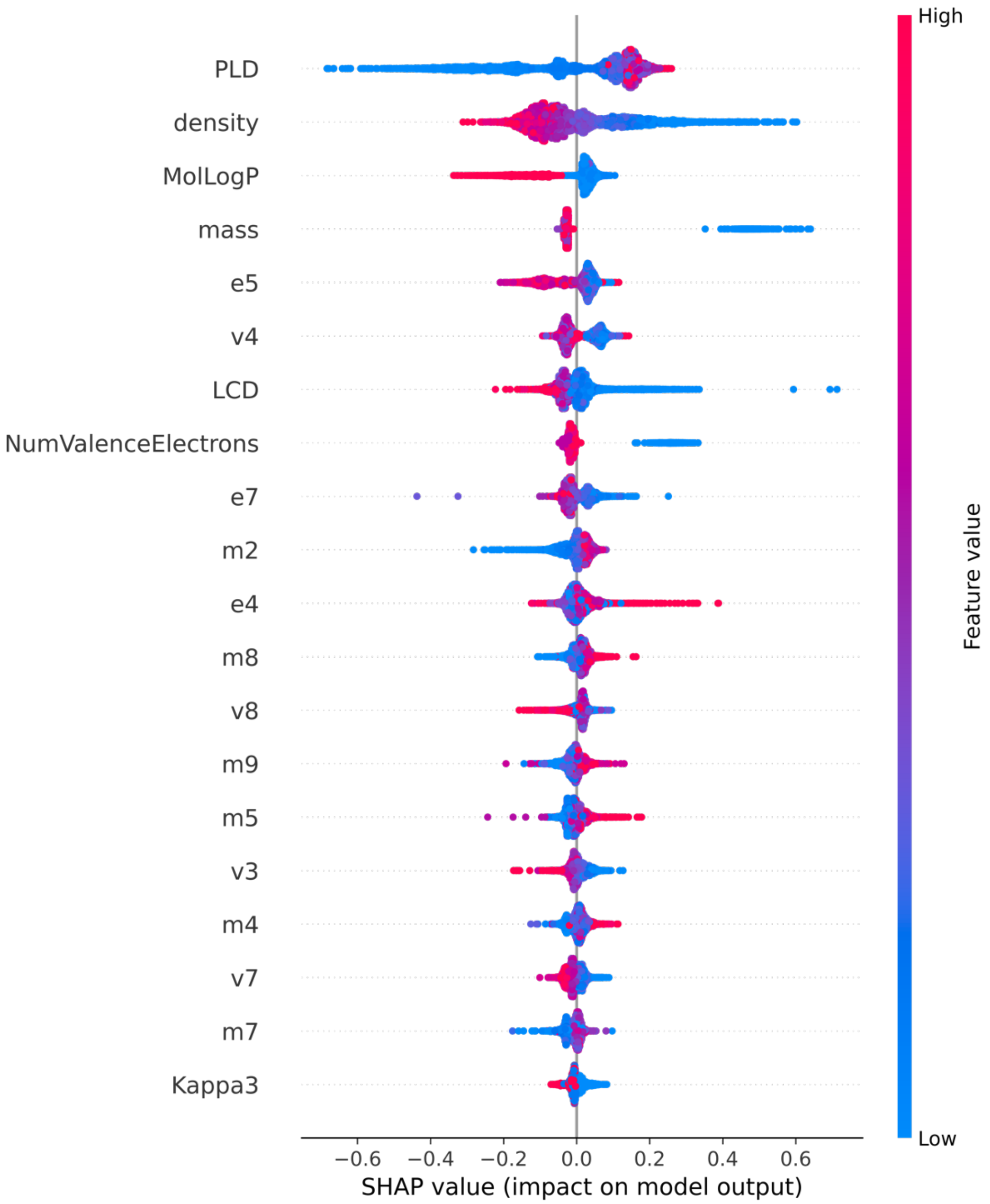

Figure S9. Shapley values summary for the top 20 features in the GBR model. The markers are color coded by each feature value.

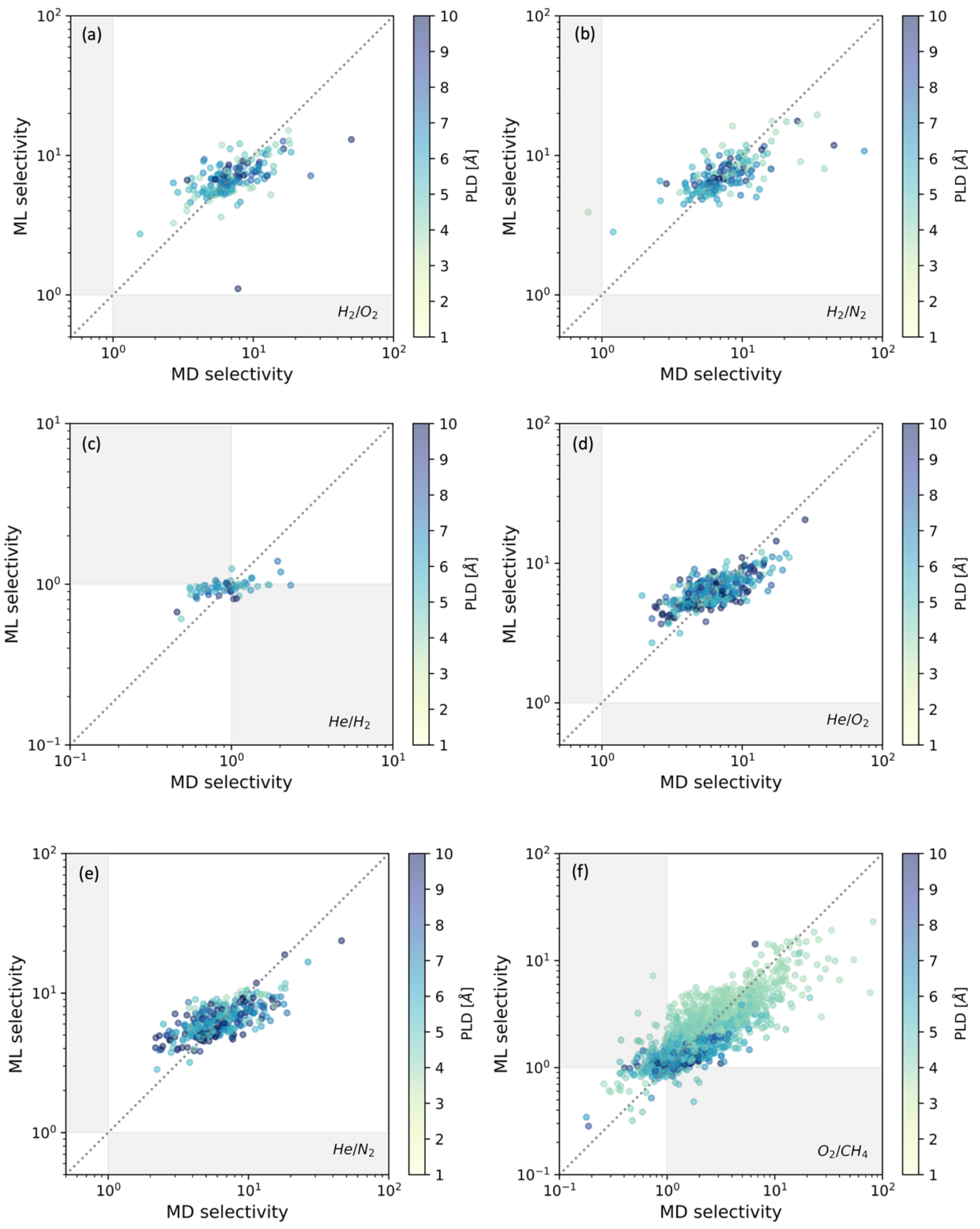

Figure S10. Parity plot of ideal diffusion selectivity of (a) H_2/O_2, (b) H_2/N_2, (c) He/H_2, (d) He/N_2, (e) He/N_2, and (f) O_2/CH_4 calculated form the MD simulations (horizontal axes) and the ML predictions (vertical axes). The data points are color coded based on the PLD of each MOF. Shadings in light grey indicate cases where the MD and ML make qualitatively different predictions for which the molecule in the mixture is favorably diffused.

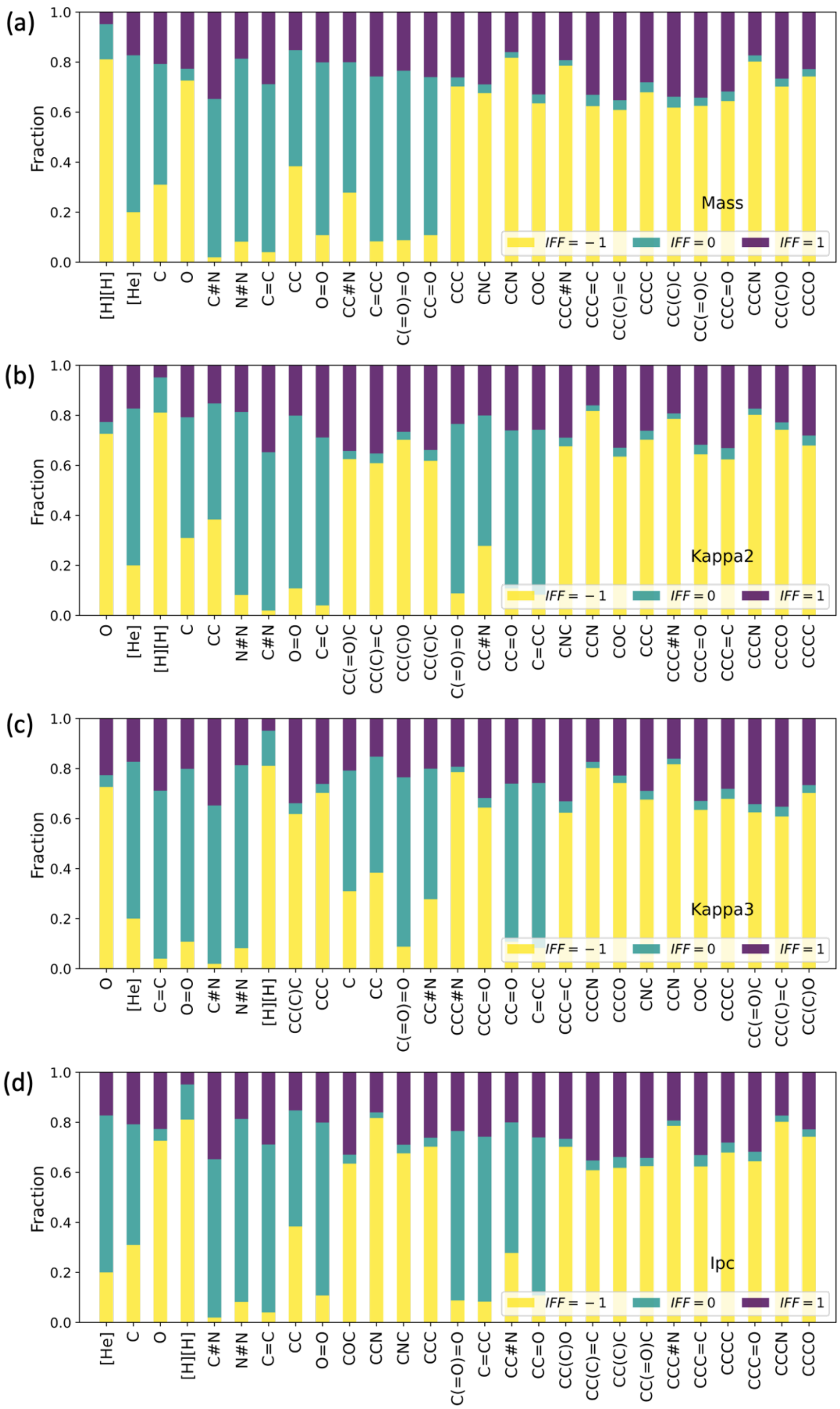

Figure S11. Distribution of the predicted IFF for each molecule. Molecules on the horizontal-axis are sorted by ascending value of (a) Mass, (b) Kappa2, (c) Kappa3, and (d) Ipc.

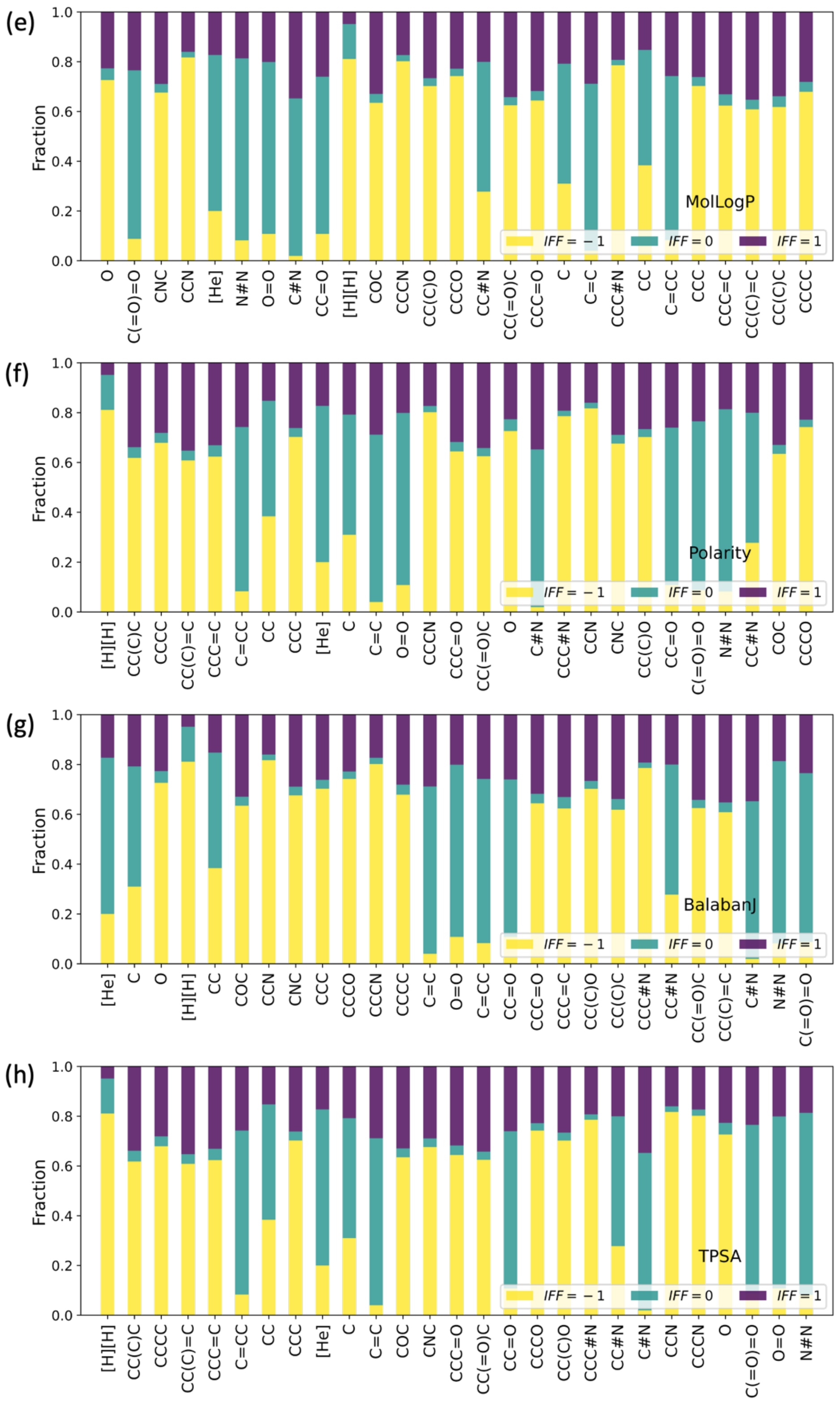

Continued. Molecules on the horizontal-axis are sorted by ascending value of (e) MolLogP, (f) polarity (g) BalabanJ, and (h)TPSA.

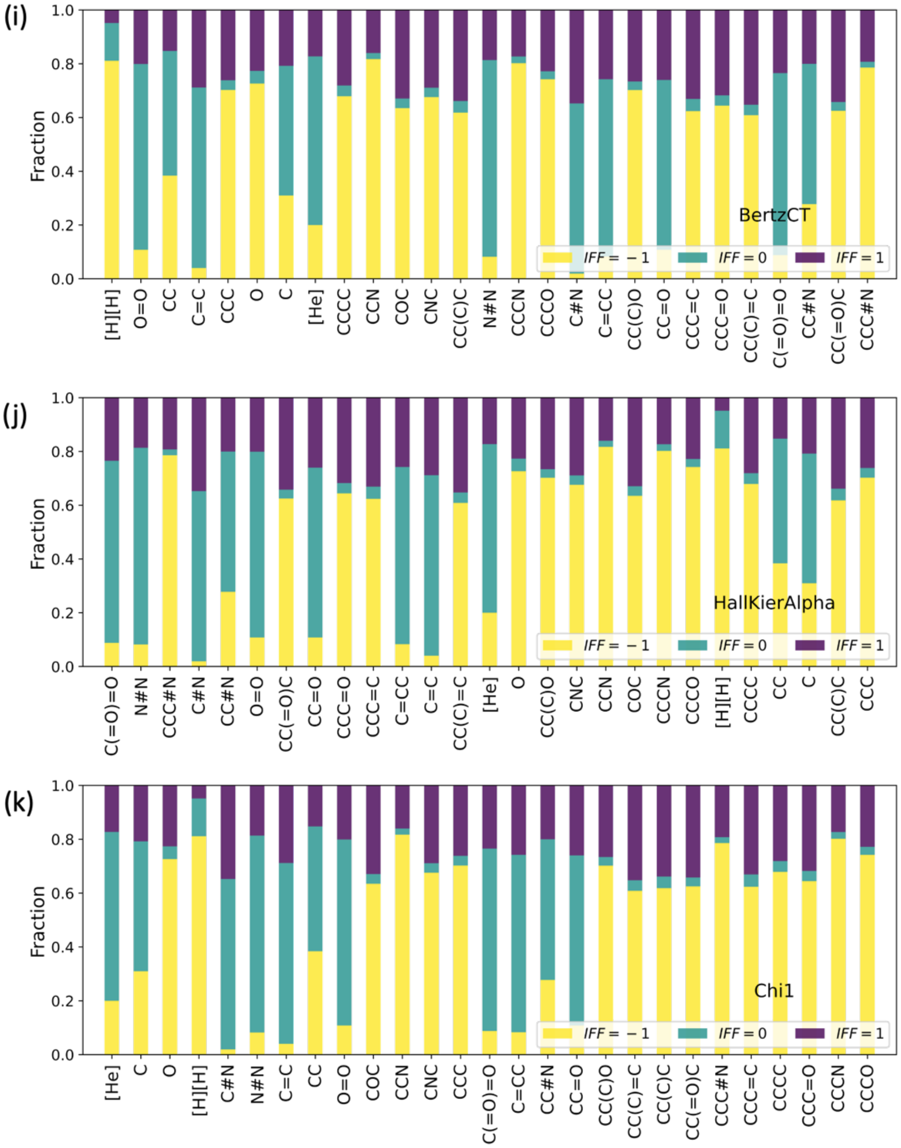

(i)
Fraction
BertzCT
IFF = −1
IFF = 0
IFF = 1
[H][H]
O=O
CC
C=C
CCC
O
C
[He]
CCCC
CCN
COC
CNC
CC(C)C
N#N
CCCN
CCCO
C#N
C=CC
CC(C)O
CC=O
CCC=C
CCC=O
CC(C)=C
C(=O)=O
CC#N
CC(=O)C
CCC#N
(j)
Fraction
HallKierAlpha
IFF = −1
IFF = 0
IFF = 1
C(=O)=O
N#N
CCC#N
C#N
CC#N
O=O
CC(=O)C
CC=O
CCC=O
CCC=C
C=CC
C=C
CC(C)=C
[He]
O
CC(C)O
CNC
CCN
COC
CCCN
CCCO
[H][H]
CCCC
CC
C
CC(C)C
CCC
(k)
Fraction
Chi1
IFF = −1
IFF = 0
IFF = 1
[He]
C
O
[H][H]
C#N
N#N
C=C
CC
O=O
COC
CCN
CNC
CCC
C(=O)=O
C=CC
CC#N
CC=O
CC(C)O
CC(C)=C
CC(C)C
CC(=O)C
CCC#N
CCC=C
CCCC
CCC=O
CCCN
CCCO

Continued. Molecules on the horizontal-axis are sorted by ascending value of (i) BertzCT, (j) HallKierAlpha, and (k) Chi1.

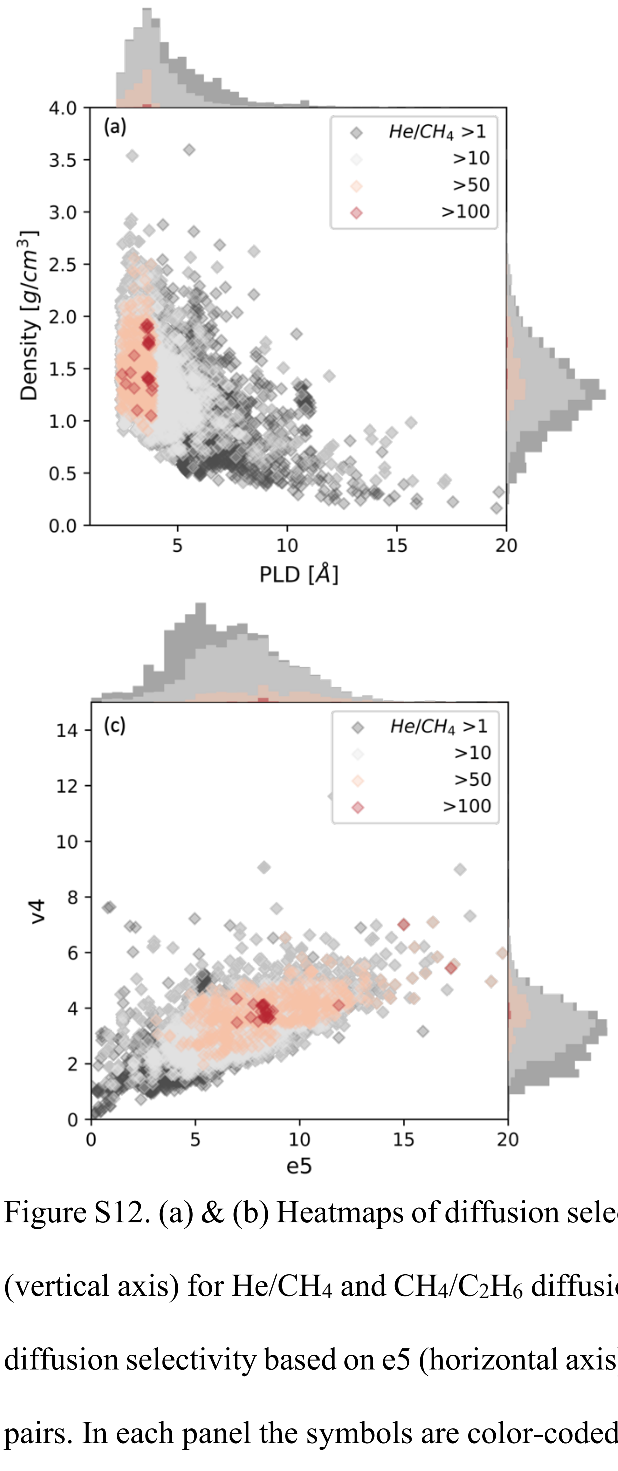

Figure S12. (a) & (b) Heatmaps of diffusion selectivity based on PLD (horizontal axis) and density (vertical axis) for He/CH_4 and CH_4/C_2H_6 diffusion selectivity, respectively; (c) & (d) Heatmaps of diffusion selectivity based on e5 (horizontal axis) and v4 (vertical axis) for the same two molecular pairs. In each panel the symbols are color-coded by the selectivity values, the molecules pairs and corresponding gradient are labeled at the top right corner of each subplot.
